# Combined genomic, transcriptomic, and metabolomic analyses provide insights into chayote (*Sechium edule*) evolution and fruit development

**DOI:** 10.1038/s41438-021-00487-1

**Published:** 2021-01-31

**Authors:** Anzhen Fu, Qing Wang, Jianlou Mu, Lili Ma, Changlong Wen, Xiaoyan Zhao, Lipu Gao, Jian Li, Kai Shi, Yunxiang Wang, Xuechuan Zhang, Xuewen Zhang, Fengling Wang, Donald Grierson, Jinhua Zuo

**Affiliations:** 1 Key Laboratory of Vegetable Postharvest Processing, Ministry of Agriculture, Beijing Key Laboratory of Fruits and Vegetable Storage and Processing, Key Laboratory of Biology and Genetic Improvement of Horticultural Crops (North China) of Ministry of Agriculture, Key Laboratory of Urban Agriculture (North) of Ministry of Agriculture, The Collaborative Innovation Center of Cucurbits Crops, Beijing Vegetable Research Center, Beijing Academy of Agriculture and Forestry Sciences, Beijing, 100097 China; 2 College of Food Science and Technology, Hebei Agricultural University, Baoding, 071001 China; 3 Beijing Advanced Innovation Center for Food Nutrition and Human Health, Beijing Technology and Business University, Beijing, 100048 China; 4 Department of Horticulture, Zhejiang University, Hangzhou, 310058 China; 5 Beijing Academy of Forestry and Pomology Sciences, Beijing Academy of Agriculture and Forestry Sciences, Beijing, 100093 China; 6 Biomarker Technologies Corporation, Beijing, 101300 China; 7 School of Biosciences, University of Nottingham, Sutton Bonington Campus, Loughborough, Leicestershire LE12 5RD UK

**Keywords:** Next-generation sequencing, Comparative genomics

## Abstract

Chayote (*Sechium edule*) is an agricultural crop in the Cucurbitaceae family that is rich in bioactive components. To enhance genetic research on chayote, we used Nanopore third-generation sequencing combined with Hi–C data to assemble a draft chayote genome. A chromosome-level assembly anchored on 14 chromosomes (N50 contig and scaffold sizes of 8.40 and 46.56 Mb, respectively) estimated the genome size as 606.42 Mb, which is large for the Cucurbitaceae, with 65.94% (401.08 Mb) of the genome comprising repetitive sequences; 28,237 protein-coding genes were predicted. Comparative genome analysis indicated that chayote and snake gourd diverged from sponge gourd and that a whole-genome duplication (WGD) event occurred in chayote at 25 ± 4 Mya. Transcriptional and metabolic analysis revealed genes involved in fruit texture, pigment, flavor, flavonoids, antioxidants, and plant hormones during chayote fruit development. The analysis of the genome, transcriptome, and metabolome provides insights into chayote evolution and lays the groundwork for future research on fruit and tuber development and genetic improvements in chayote.

## Introduction

Chayote (*Sechium edule*) is a diploid perennial herbaceous climbing plant with 28 chromosomes (2*n* = 2*x* = 28)^1^ that belongs to the Cucurbitaceae family^2^. The chayote fruit is a gourd that is consumed as a vegetable, also called vegetable pear, chuchu, pear squash, or sayote^3,4^, and the tubers are also utilized as food source. Chayote is believed to have originated in Mexico, where it was first cultivated approximately 500 years ago^5,6^. It is commonly cultivated in tropical and subtropical areas, such as Brazil, India, Costa Rica, China, and Mexico, and is a significant commercial crop worldwide^7,8^. Chayote contains abundant bioactive compounds, such as phenolics, flavonoids, carotenoids, and bioactive polysaccharides^9–11^ in the fruit, leaves, tubers, and stems^4,12^, and has potential for the treatment of hypertension, diabetes, and inflammation, as well as other pharmacological applications^2,13,14^.

In recent years, reports about chayote have been mainly related to the function, compositions, and applications of its fruit, stems, leaves, and tubers^4,9,15,16^. Although there has been extensive exploration of the genomes within the Cucurbitaceae family, such as *Cucumis sativus* (2*n* = 2*x* = 14, 226.2 Mb)^17^, *Cucumis melo* (2*n* = 2*x* = 24, 398.57 Mb)^18^, *Citrullus lanatus* (2*n* = 2*x* = 22, 353.5 Mb)^19^, *Cucurbita pepo* (2*n* = 2*x* = 20, 263 Mb)^20^, *Luffa cylindrica* (2*n* = 2*x* = 26, 656.19 Mb)^21^, *Lagenaria siceraria* (2*n* = 2*x* = 22, 313.4 Mb)^22^, and *Benincasa hispida* (2*n* = 2*x* = 24, 913 Mb)^23^, there is no complete genome of chayote available at present^24^, and there have only been a few studies on methods for chayote DNA extraction^25^ and on the genes expressed in chayote fruit^26,27^.

Genome sequences provide resources for studies on evolution, genetic variation and traits for crop improvement^28^. To advance research on chayote, we assembled a draft genome using Nanopore and Hi–C data. Repeat sequences were identified, the functions of protein-coding genes were annotated, and expanded gene families were identified. Comparative genomics analysis indicated that of the species we studied, chayote is most closely related to snake gourd. Evidence for a paleoploidization (whole-genome duplication (WGD)) event in chayote was detected. The genes and mechanisms involved in fruit texture, pigment, flavor, plant hormones, and antioxidant properties were investigated. The chromosome-scale assembly allows a better understanding of evolution in Cucurbitaceae and provides insights for trait modification and breeding.

## Results

### Genome sequencing, assembly, and evaluation

**Fig. 1:**
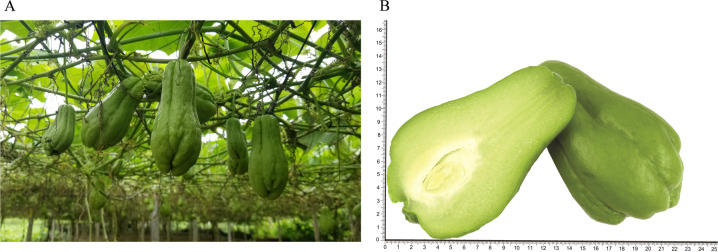
Chayote fruits. **A** Fruiting chayote plants grown in Jianshui, Yunnan Province. The fruit photographed was approximately 10 × 18 cm; **B** longitudinal section of mature chayote fruits on the market

Two libraries were built for the Illumina sequencing platform, and 39.01 Gb of high-quality data were obtained after filtration, with a total sequencing depth of approximately 55 with Q20 and Q30 percentages of 97.09 and 92.02, respectively. From the total kmer number and average kmer depth, the chayote genome length was estimated to be 710.23 Mb (Fig. S1). The proportion of repeat sequences was estimated to be approximately 62.93%, the heterozygosity rate was approximately 0.03%, and the GC content of the genome was approximately 40% (Fig. 1).

Approximately, 100.56 Gb of raw data was obtained. After data quality control (QC), the clean data volume was 91.97 Gb, representing a 151× sequencing depth. The clean data contained 4,155,091 reads with a read N50 of 29.68 kb and average read length of 22.13 kb (Table 1). Finally, a total of 608.17 Mb of genome sequence in 356 contigs (N50 of 10.09 Mb) with 38.71% GC content was obtained (Table 1). A total of 99.4% clean reads were mapped to the Nanopore reference genome. The CEGMA v2.5 database^29^ contains 458 conserved core genes and 248 highly conserved genes from eukaryotes. The chayote assembled genome contained 445 core eukaryotic genes (CEGs) (97.16%) and 223 highly conserved CEGs (89.92%). Benchmarking universal single-copy orthologs (BUSCO) v2.0 software^30^ was used to assess the integrity of the genome assembly, covering 2121 conserved core genes, and 2028 (95.62%) complete BUSCOs were found, which contained 1780 (83.92%) single-copy, 248 (11.69%) duplicated, 23 (1.08%) fragmented genes and 70 (3.30%) missing BUSCOs (Table 1).

**Table 1:** Statistics of genome sequencing, Hi–C assembly and gene assessment in BUSCO

Parameter	Value
*Nanopore sequencing*	
Contig number	356
Contig N50 (Mb)	10.09
Genome size (Mb)	608.17
*Hi–C assembly*	
Scaffold number	103
Scaffold N50 (Mb)	46.56
Contig number	473
Contig N50 (Mb)	8.40
Genome size (Mb)	606.42
*BUSCO evaluation*	
Complete BUSCOs	2028 (95.62%)
Complete and single-copy BUSCOs	1780 (83.92%)
Complete and duplicated BUSCOs	248 (11.69%)
Fragmented BUSCOs	23 (1.08%)
Missing BUSCOs	70 (3.30%)
Total lineage BUSCOs	2121

### Chromosome-level assembly of Hi–C

**Fig. 2:**
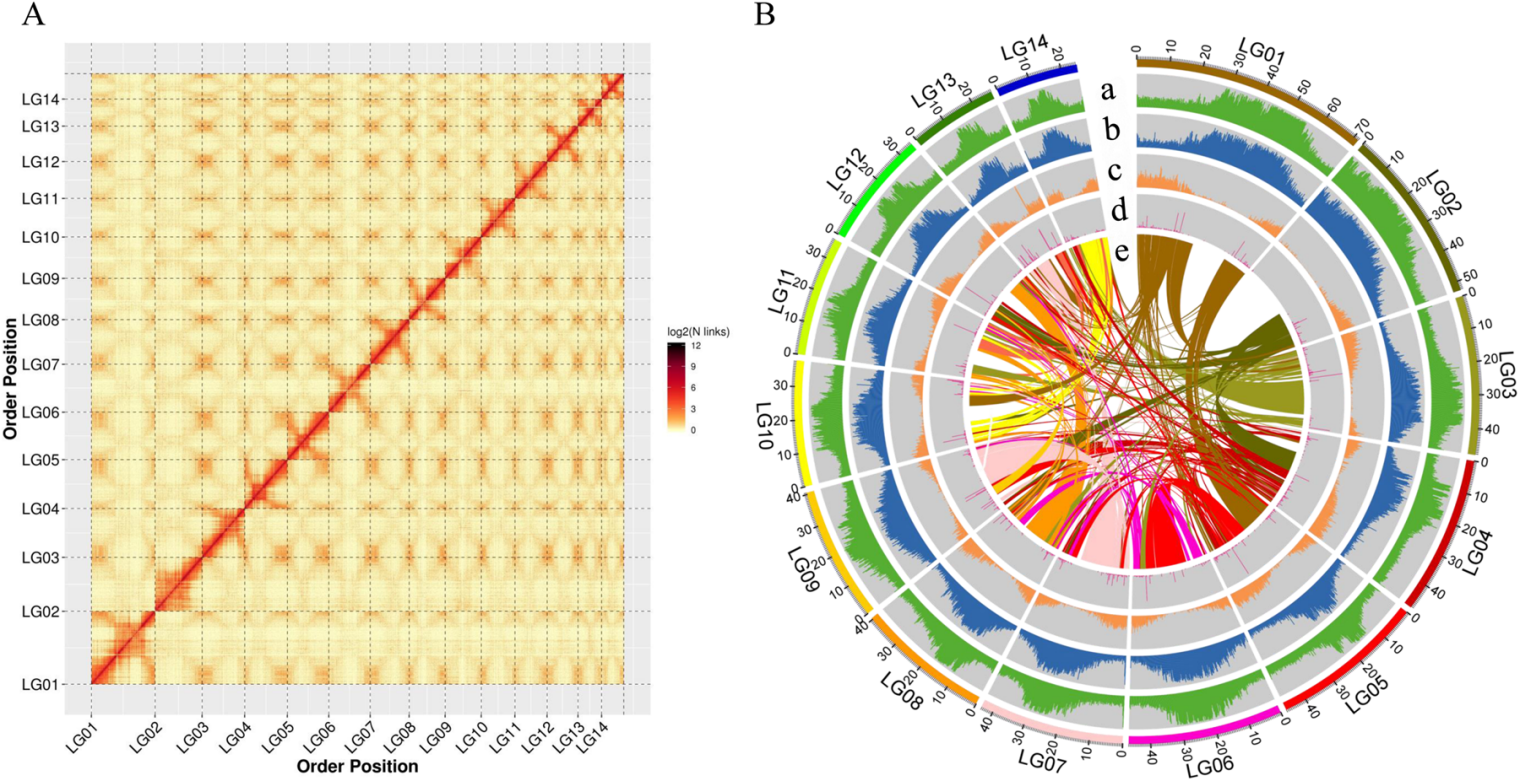
Chayote genome information. **A** Hi–C heat map of chromosome interactions; **B** overview of chayote genomic features. **a** GC content; **b** repeat sequence coverage; **c** gene density; **d** noncoding RNA density; and **e** chromosome collinearity

Hi–C technology is derived from chromosome conformation capture technology combined with high-throughput sequencing. It evaluates the interactions of chromosomes in three-dimensional space by capturing and sequencing the interactions between DNA fragments in chromosomes, information that can contribute to genome assembly. Ultimately, 606.42 Mb of genome sequence was anchored on 14 chromosomes by Hi–C assembly and manual adjustment, accounting for 97.04% of all genome sequence (Table S1). After correction of chromosome order and direction, a genome sequence length of 598.48 Mb was obtained, accounting for 98.69% of the total sequence length, with contig N50 and scaffold N50 of 8.40 and 46.56 Mb, respectively (Tables 1 and S2). The Hi–C heat map (Fig. 2A) shows the effectiveness of the genome assembly; an overview of the genome assembly is shown in Fig. 2B.

### Genome annotation analysis

**Fig. 3:**
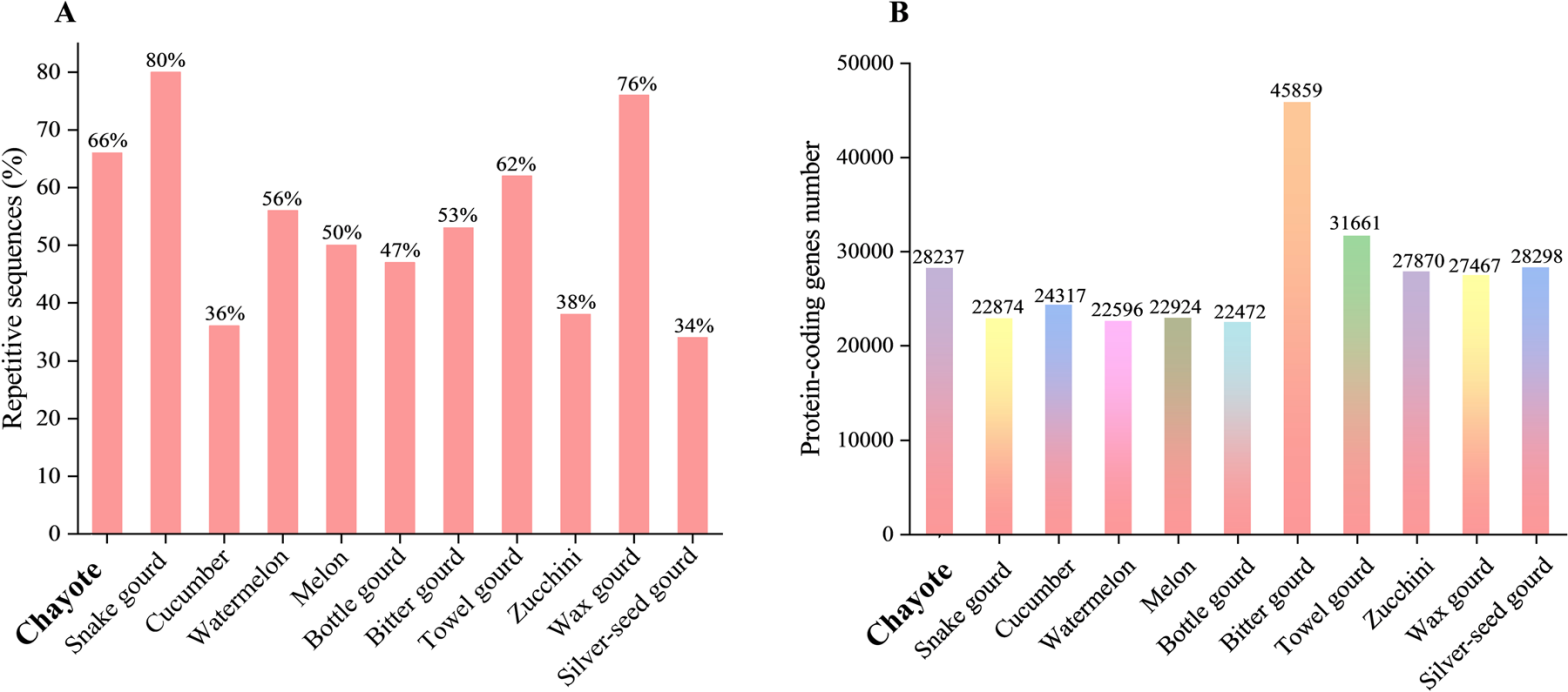
Comparison of chayote with other Cucurbitaceae genome assemblies. **A** Percentage of repetitive sequences in chayote and ten other Cucurbitaceae; **B** comparison of numbers of protein-coding genes in chayote and ten other Cucurbitaceae (snake gourd^37^, cucumber^17^, watermelon^49^, melon^18^, bottle gourd^22^, bitter gourd^50^, towel gourd^21^, zucchini^20^, wax gourd^23^, silver-seed gourd^53^). The colors in **B** indicate different species

In total, 401.08 Mb of repetitive sequences were identified by analysis of the genome database and structure predictions, representing 65.94% of the whole genome. Long terminal repeats (LTRs) accounted for the highest proportion (36.96%) of the genome, including 9.11% Copia-LTRs and 27.85% Gypsy-LTRs (Table S3). Ab initio predictions, homology-based predictions and RNA-seq fragments were used to predict the genetic structure (Fig. S2), and 28,237 protein-coding genes were detected (Table S4). The average numbers of exons and introns per gene were 5.70 and 4.70, respectively (Table S5). The noncoding RNAs identified included 101 microRNAs, 1873 tRNAs and 298 rRNAs. Through BLA comparison and GeneWise, 1085 pseudogenes were found. Kyoto encyclopedia of genes and genomes (KEGG), KOG (eukaryotic orthologous groups), and gene ontology (GO) were used for functional annotation analysis and produced results for 97.28% of the assembled genome, including GO (53.19%), KEGG (32.31%), KOG (52.58%), TrEMBL (97.16%), and Nr (97.22%) (Table 2). Only 2.72% of the genome sequence was unannotated. Comparisons of repetitive sequence percentages and protein-coding gene numbers in chayote and ten other Cucurbitaceae are summarized in Fig. 3.

**Table 2:** Genome annotation statistics

Parameter	Number	Percentage (%)
Total repetitive sequences	957,675	65.94
Protein-coding genes	28,237	–
miRNA	101	–
rRNA	298	–
tRNA	1873	–
Pseudogenes	1085	–
All functional annotations	27,469	97.28
GO_annotations	15,018	53.19
KEGG_annotations	9124	32.31
KOG_annotations	14,847	52.58
TrEMBL_annotations	27,435	97.16
Nr_annotations	27,451	97.22
Unannotated	768	2.72

### Comparative genomics analysis

**Fig. 4:**
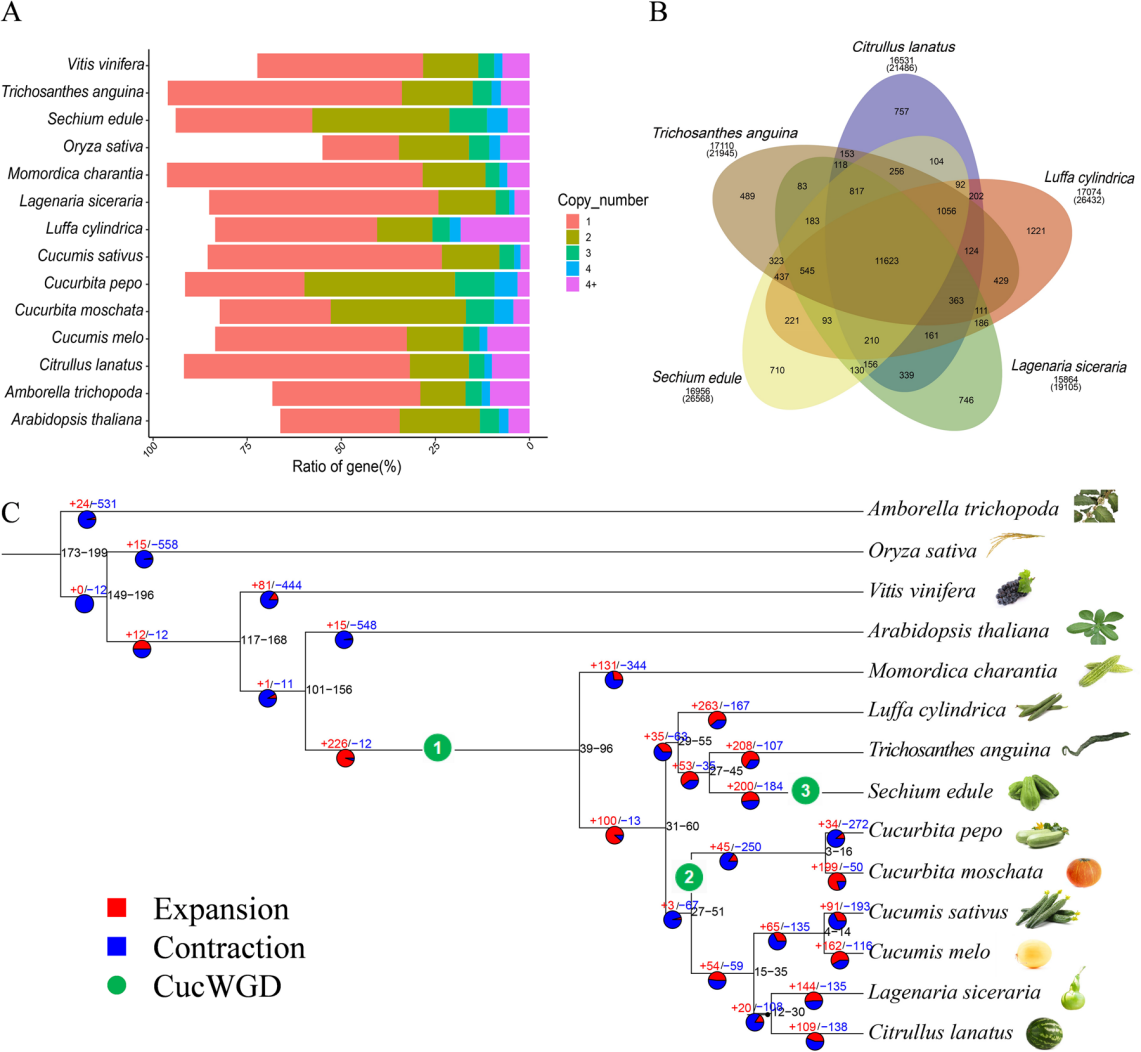
Analysis of gene families and phylogeny of chayote and other related plant genomes. **A** Gene copy number distribution in chayote and 13 other sequenced plant genomes; **B** Venn diagram representing the clusters of gene families in chayote and four related plants (*Trichosanthes anguina, Luffa cylindrica, Citrullus lanatus, Lagenaria siceraria*); **C** phylogenetic tree and gene family expansions/contractions in chayote and 13 other species (*Cucumis sativus*, *Citrullus lanatus*, *Luffa cylindrica*, *Lagenaria siceraria*, *Cucurbita moschata*, *Trichosanthes anguina*, *Momordica charantia*, *Cucurbita pepo*, *Cucumis melo, Arabidopsis thaliana*, *Vitis vinifera*, *Amborella trichopoda*, and *Oryza sativa*) based on a concatenated alignment of 832 single-copy protein sequences. The tree is rooted with *A. trichopoda* as the outgroup. Red represents expanded gene families, blue represents contracted gene families, and green indicates Cucurbit-common whole-genome duplication (CucWGD)

To study the evolution of the chayote genome, we conducted comparative genomics analysis of 14 species, including 11 cucurbits (*S. edule*, *C. sativus*, *C. lanatus*, *L. cylindrica*, *L. siceraria*, *Cucurbita moschata*, *Trichosanthes anguina*, *Momordica charantia*, *C. pepo*, *B. hispida*, *C. melo*) and *Arabidopsis thaliana*, *Vitis vinifera*, and *Amborella trichopoda*. A total of 35,291 gene families were analyzed, representing 2,854 common gene families, and there were 134 specific gene families in the chayote genome (Fig. S3). These orthogroup gene statistics were calculated for each species, and chayote contained 36.3% single-copy genes and 36.4% two-copy genes (Fig. 4A). Cluster analysis of gene families was performed for *S. edule, T. anguina, L. cylindrica, C. lanatus* and *L. siceraria* (Fig. 4B), and GO and KEGG enrichment were used to analyze the specific gene families in chayote (*S. edule)* (Fig. S4). The unique genes were related to binding, catalytic activity, and metabolic and cellular processes. KEGG analysis showed that they were enriched in mismatch repair, DNA replication, homologous recombination, and nucleotide excision repair.

We constructed an evolutionary tree from 832 single-copy protein sequences (Fig. 4C) and found that *S. edule* and *T. anguina* (snake gourd) had the closest evolutionary relationship (27–45 Mya) and were estimated to have separated from *L. cylindrica* at 29–55 Mya. We predicted 184 contracted (Fig. S5) and 200 expanded (Fig. S6) gene families (Fig. 4C), which, according to GO and KEGG enrichment annotations, were enriched for glycosaminoglycan degradation, stilbenoid, diarylheptanoid, flavonoid and gingerol biosynthesis, wax, cutin, suberin metabolism, tryptophan metabolism, and phenylpropanoid biosynthesis genes. The expanded gene families classified by KEGG pathway identified genes for linoleic acid metabolism, phenylalanine metabolism, alpha-linolenic acid metabolism and diterpenoid biosynthesis, starch and sucrose metabolism, pentose and glucuronate interconversions, and phenylpropanoid biosynthesis.

### Collinearity analysis and WGD

**Fig. 5:**
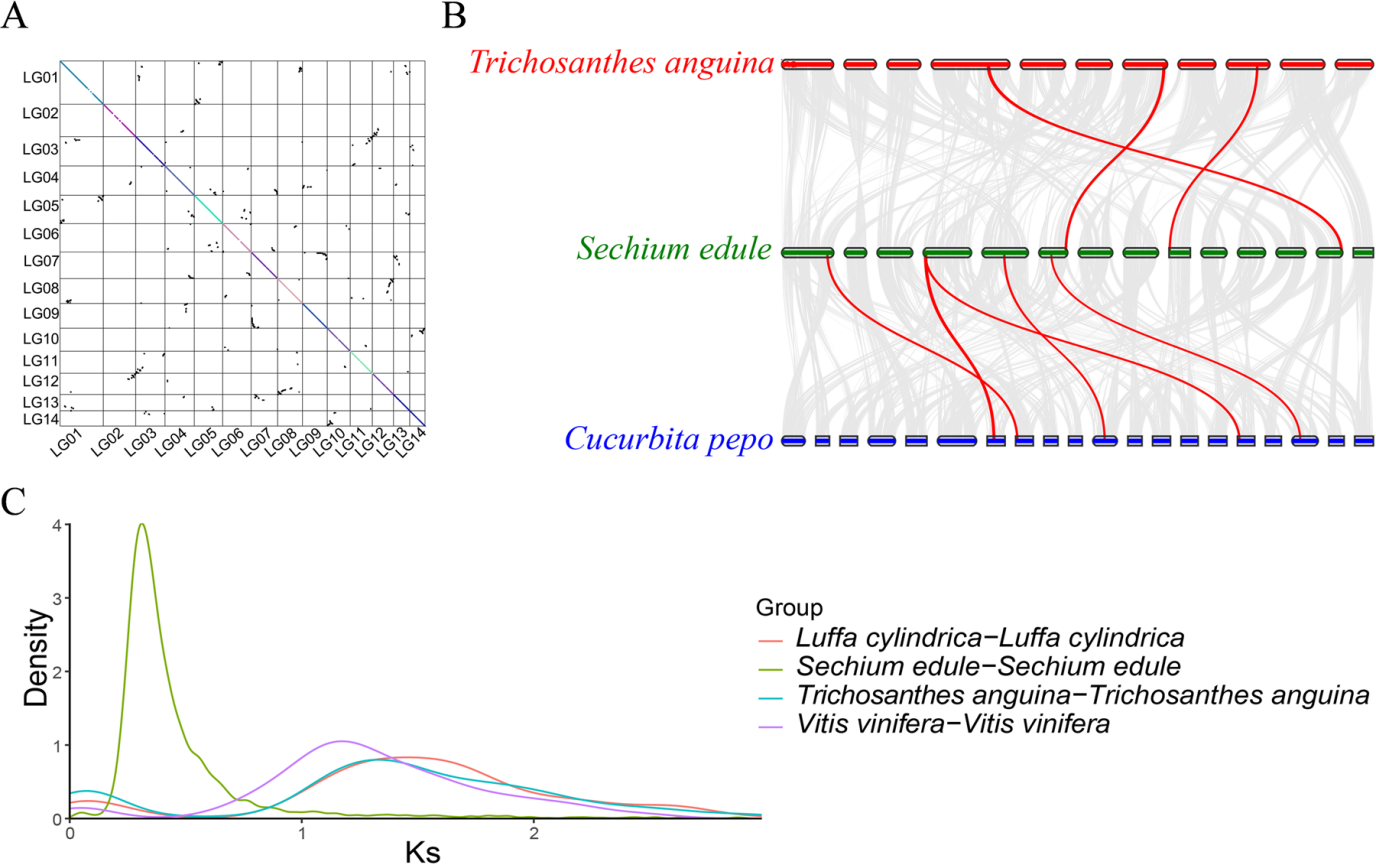
WGD analysis of chayote. **A** Dot plots of chayote genome paralogs; **B** chromosome collinearity analysis among chayote, snake gourd and pumpkin; red lines represent genes involved in fruit development; **C** Ks distribution of chayote and other related plant species

Paralogous genes were identified in chayote and pumpkin through genome collinearity analysis. This identified 41,018 collinear genes, accounting for 73.11% of the total gene number (56,010) (Fig. 5), which suggested a high degree of conserved gene order in the two species, although there were significant rearrangements. The collinearity analysis identified a mass of synonymous gene blocks in chayote and pumpkin. The comparison of chayote and pumpkin indicated that there have been far more interchromosomal rearrangement events than between chayote and snake gourd (72.2%) (Fig. 5). With the exception of *peroxidase 45-like* (EVM0001323.1), the genes indicated in Fig. 5B are related to phytohormones, such as *auxin response factor 17* (EVM0017908.1), *auxin response factor 9* (EVM0019236.1), *auxin-induced protein AUX22-like* (EVM0027070.1), *1-aminocyclopropane-1-carboxylate synthase* (EVM0000580.1), *1-aminocyclopropane-1-carboxylate oxidase 5-like* (EVM0026245.1), and *ethylene-responsive transcription factor 3-like* (EVM0008801.1), which were present in all three fruit species.

WGD events are of great significance in generating species diversity during evolution. It can be deduced from the Ks and 4DTv distribution, which in chayote had peaks at approximately 0.314 and 0.102 (Figs. 5 and S7), which indicated that a paleoploidization (WGD) event occurred recently, as proposed by others^31^. These WGD events are shown by dot plots of paralogs in Fig. 5A. An ancient WGT (*γ*) event (approximately 130–150 Mya)^32,33^ that occurred after the divergence of monocotyledons and dicotyledons^34^, was confirmed for grape, towel gourd, and snake gourd by the peak shown in Fig. 5C. From the divergence date of recent WGDs, such as those in cucurbita^20^ (4DTv = 0.12, 30 ± 4 Mya) and soybean^35^ (4DTv = 0.057, 13 Mya), we estimated that the chayote WGD event occurred 25 ± 4 Mya (*T* = *D*/2*μ*). According to the evolutionary tree (Fig. 4C), chayote diverged from snake gourd (27–45 Mya) and towel gourd (29–55 Mya), which suggested that the WGD of chayote occurred after its differentiation from towel gourd and snake gourd. No WGD event was observed previously for towel gourd^21,36^ or snake gourd^37^, consistent with our results.

### Transcription and metabolism in developing chayote fruit

**Fig. 6:**
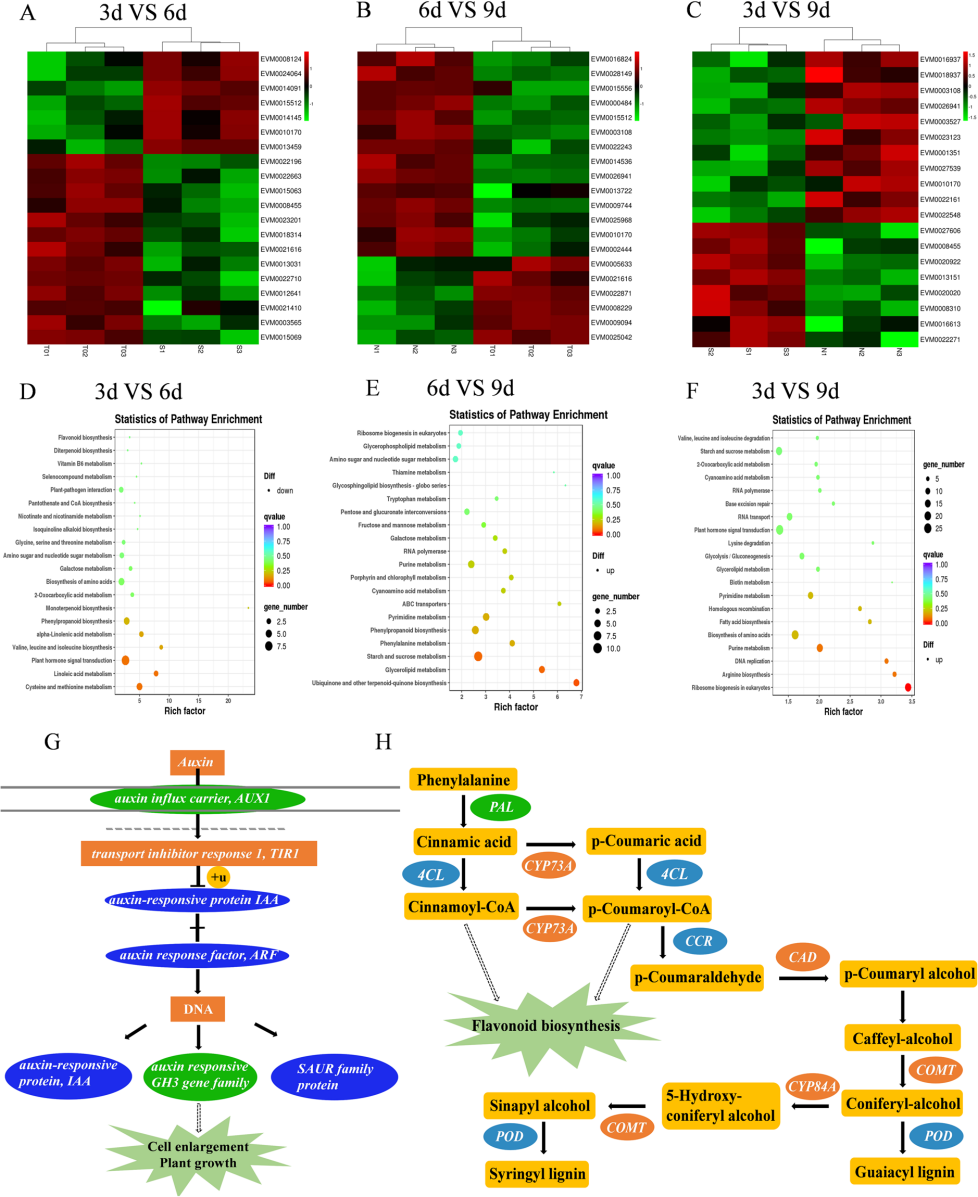
Transcriptome KEGG annotation and enrichment map for genes expressed during chayote fruit development. **A** Heat map of major genes differentially expressed in fruit (3 days vs. 6 days); **B** heat map of major genes differentially expressed in fruit (6 days vs. 9 days); **C** heat map of major genes differentially expressed in fruit (3 days vs. 9 days); **D** KEGG annotation of transcripts decreased from 3 days to 6 days fruit; **E** KEGG annotation of transcripts increased from 6 days to 9 days fruit; **F** KEGG annotation of transcripts increased from 3 days to 9 days fruit; **G** changes in gene expression related to plant hormone signal transduction pathways in 3 days vs. 9 days; green represents decreased, blue represents decreased and increased; **H** changes in expression of gene of phenylpropanoid biosynthesis in 6 days vs. 9 days, orange represents increased, green represents decreased, blue represents decreased and increased, yellow represents unchanged

**Fig. 7:**
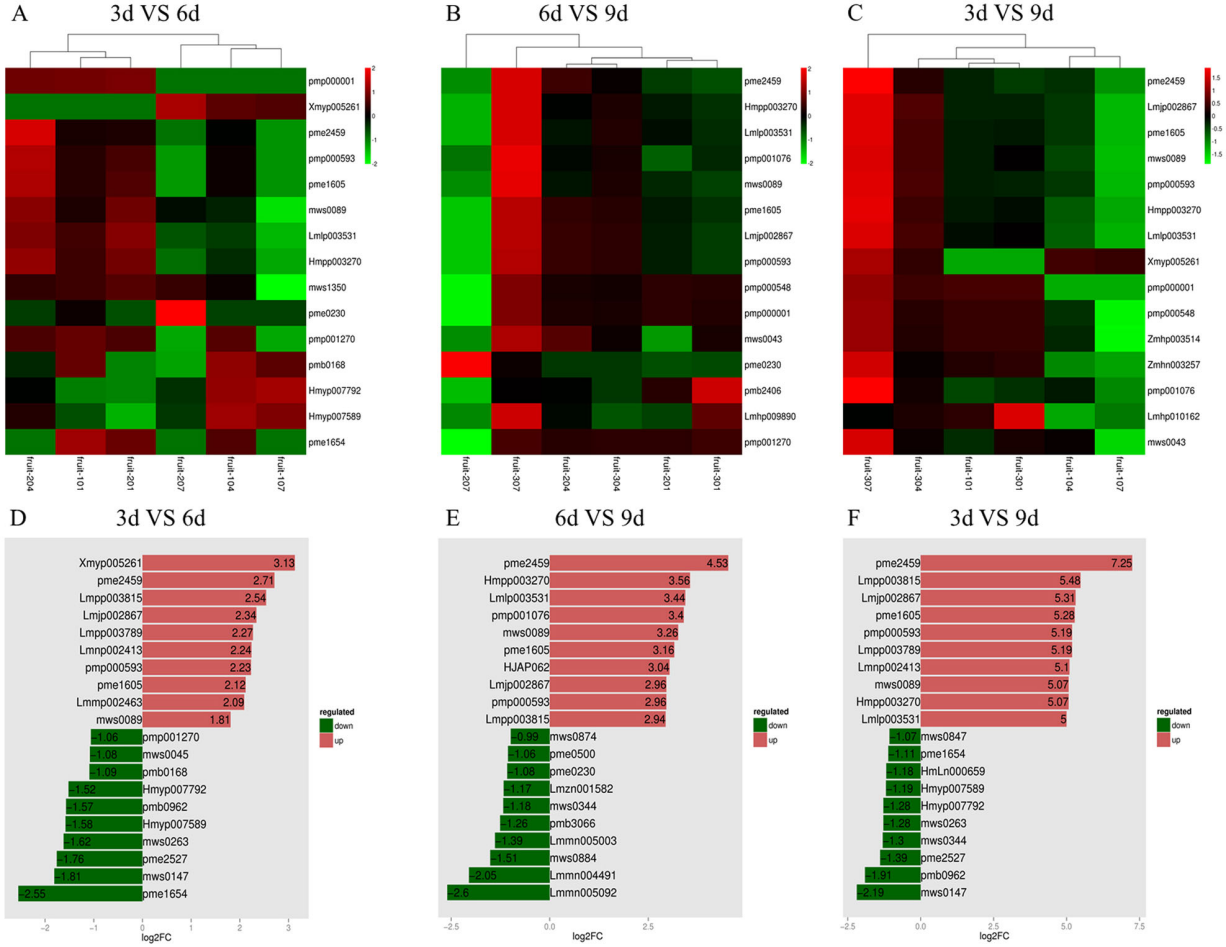
Changes in metabolism during fruit development. **A** Heat map showing major changes in metabolites in 3 days vs. 6 days fruit; **B** heat map showing major changes in metabolites in 6 days vs. 9 days fruit; **C** heat map showing major changes in metabolites in 3 days vs. 9 days fruit; **D** top 20 greatest fold-changes in metabolites in the comparison of in 3 days vs. 6 days; **E** top 20 greatest fold-changes in metabolites in the comparison of 6 days vs. 9 days; **F** top 20 greatest fold-changes in metabolites in the comparison of 3 days vs. 9 days

Transcriptomics and metabolomics were used to study changes during fruit development. Correlations between samples in transcriptomics and orthogonal partial least squares-discriminant analysis (OPLS-DA) assessment of metabolomics were checked and compared with differential expression analysis (Fig. S8). A total of 385 differentially expressed genes (DEGs) were identified in the 3 days vs. 6 days comparison (Fig. 6), consisting of 118 upregulated genes and 267 downregulated genes; similarly, 34 different metabolites were identified as differentially abundant, including 12 that decreased and 22 that increased, and these were annotated with KEGG and GO terms (Figs. 7 and S9, Tables S6 and S7). In the comparison of 6 days vs. 9 days, a total of 57 different metabolites and 1033 DEGs were identified, consisting of 464 upregulated genes and 569 downregulated genes and 8 decreased and 49 increased metabolites (Fig. 7, Tables S6 and S7, Fig. S10). In the 3 days vs. 9 days comparison, 48 different metabolites were found, of which 40 were upregulated and 8 were downregulated (Fig. 7, Tables S6 and S7). A total of 3281 DEGs, consisting of 1606 upregulated and 1675 downregulated genes, were clustered and annotated with KEGG and GO terms (Figs. S11 and S12).

Among the three comparison groups, transcripts of genes involved in plant hormone synthesis and response were markedly changed in chayote fruit. *Auxin-induced protein 22D-like* (*AUX22D/22B/22*) and *auxin-responsive protein IAA13-like* (*IAA13/9*) were obviously upregulated in the 3 days vs. 6 days comparison. In the 6 days vs. 9 days comparison, transcripts of *auxin-responsive protein SAUR50-like* (*SAUR50*) increased 23-fold; *auxin-responsive protein IAA13-like* (*IAA13/IAA11*), *auxin-induced protein 22B-like* (*AUX22B*), and *gibberellin 20 oxidase 1-like* (*GB*) showed similar trends, while *1-aminocyclopropane-1-carboxylate synthase 7* (*ACS7*), *1-aminocyclopropane-1-carboxylate oxidase homolog 6* (*ACO6*), and *abscisic acid-insensitive 5-like protein 6* (*ABA5*) were downregulated. In the 3 days vs. 9 days comparison group, transcript levels of *ACS7*, *ACO6*, and *ABA5* also decreased. Genes encoding other enzymes associated with plant hormones were upregulated, such as *AUX22B/AUX22D*, *IAA11/IAA13/IAA14/IAA21*, and *SAUR50*. Overlaps between these comparison groups provide information about the development of chayote fruit. *AUX22B, AUX22D*, and *IAA13*, which are auxin growth-related factors, increased, and a role for auxin is indicated in Fig. 6E. Auxin is linked to *transport inhibitor response 1* (*TIR1*), which can direct polyubiquitylation^38^. When auxin levels increase, *AUX/IAA* proteins are targeted for proteasomal degradation, which is a key factor in the regulation of auxin signaling^39^. With the release of free active ARFs, the activation of target genes is promoted^40^. In addition, *auxin-responsive protein* (*IAA*), auxin-responsive *GH3* family genes and *SAUR* family proteins are used to regulate fruit growth and development. *IAA* and *GH3* gene families were up and downregulated, respectively, and *SAUR* family proteins expression decreased from 3 to 9 days, which was correlated with chayote fruit enlargement. Transcripts for *ACS7* and *ACO6*, which together catalyze the biosynthesis of ethylene^41^, decreased from 6 to 9 days, indicating a likely reduction in ethylene production at this stage.

mRNAs for several TFs changed significantly in the comparisons between different stages of fruit development. In the 3 days vs. the 6 days comparison, *bHLH94, MYB3R-1-like* and *NAC TF* (*NAM-2*) increased, whereas ethylene-responsive TFs (*ERF109/017/4/11/ERF1B/AP2-1*), *MYB* (*MYB108/44/24*)*, MYC* (*MYC3/2*), *WRKY* (*WRKY40/46/2*), *bHLH 93*, *auxin response factor 9* (*ARF9*), *TF HBP-1b*, *GATA TF 9-like* were downregulated. The transcription of *MYC2*, which is involved in jasmonic acid signaling, decreased. In the 6 d vs 9 d comparison, transcripts for TFs, such as *bHLH* (*bHLH118/91/82/69*), *ERF* (*ERF105/98/106/RAP2-3*), *bZIP* (*bZIP11/44*), probable *WRKY* (*WRKY49/57*), and *MYB* (*MYB59/48*) were upregulated, and those for other *ERFs* (*ERF60/110/11/61/AIL1/5/6*), *MYB* (*MYB41/44/111*), *TCP* (*TCP2/17*), *bHLH149*, *PIF3*, *GTE7*, and *VOZ1* were decreased. In the 3 days vs. 9 days comparison, downregulated TFs included *WRKY* (*WRKY 22/12/7/1/34*), *PIF3/PIF5*, *MYC2*, *TGA1/TGA9*, *ERF* (*ERF11/3/4/53/60/61/106/110/113/RAV1/2/AIL1/5/6/ANT/RAP2-1/2-4/2-7*), *MYB* family TF (*PHL11*), *bHLH* (*bHLH13/112/149/30/69/74/78/93/102/108/111/1R1/20/24/30/41/44*), and *NAC1/NAC25*. The upregulated TFs included *bZIP11/bZIP14*, *ERF* (*ERF98/105/106/RAP2-10/2-11/2-1/2-3*), *MYB* family TFs (*APL/PHL4*), *NAC* (*NAM-2*), *WRKY* (*WRKY 21/23/4/57*), and *bHLH* (*bHLH110/118/143/155/51/68/78/79/82/91/3R-1/48/59*).

*bHLH* and *MYB* are significant TF families for controlling the biosynthesis of isoflavonoids and flavonoids^42^. Phenylpropanoid biosynthesis pathway was the upstream part of flavonoid biosynthesis, and the transcript levels of several of these genes changed markedly in the 6 days vs. 9 days comparison group (Fig. 6H); some of these genes were associated with the production of lignin and phenolic compounds rather than flavonoids. Through *phenylalanine ammonia-lyase* (*PAL*), cinnamic acid is converted to cinnamoyl-CoA and p-coumaroyl-CoA by *4-coumarate-CoA ligase* (*4CL*) and *trans-cinnamate 4-monooxygenase* (*CYP73A*), which has been confirmed to participate in the biosynthetic pathway leading to flavonoids^43^. Genes encoding enzymes involved in the production of alcohols, such as *cinnamoyl-CoA reductase* (*CCR*), *caffeic acid 3-O-methyltransferase* (*COMT*), and *ferulate-5-hydroxylase* (*F5H*, *CYP84A*), were upregulated. Peroxidase (POD) is involved in lignin production. The concentration of many metabolites increased, such as hispidulin, luteolin-4′-O-glucoside, kaempferol-7-O-glucoside, quercetin-3-O-glucoside, luteolin-C-rhamnosyl-glucoside, luteolin-7-O-rutinoside, kaempferol-3-O-robinobioside (biorobin), and luteolin-3’-O-glucoside in the 3 d vs 6 d comparison. Furthermore, in the 6 days vs. 9 days comparison, luteolin-7-O-glucoside (cynaroside or luteoloside) (23-fold increase), luteolin-4′-O-glucoside (11-fold increase), luteolin-3′-O-glucoside (10-fold increase), isosinensetin (10-fold increase), kaempferol-7-O-glucoside (9.5-fold increase), kaempferol-3-O-robinobioside (biorobin) (8.9-fold increase), pratensein, nobiletin, hispidulin, quercetin-3-O-rhamnoside, luteolin-7-O-rutinoside, kaempferol-3-O-neohesperidoside, luteolin-6-C-glucoside (isoorientin) and diosmetin-7-O-galactoside concentrations increased. Notable increases in metabolites from 3 to 9 days samples included luteolin-7-O-glucoside (cynaroside) (151-fold), kaempferol-3-O-neohesperidoside (39-fold), kaempferol-3-O-robinobioside (biorobin) (38-fold), luteolin-7-O-rutinoside (36-fold), luteolin-C-rhamnosyl-glucoside (34-fold), kaempferol-7-O-glucoside (33-fold), luteolin-4′-O-glucoside, luteolin-3′-O-glucoside (33-fold), quercetin-3-O-glucoside (32-fold), hispidulin (12-fold), pratensein (11-fold), isosinensetin (9-fold), diosmetin-7-O-galactoside (8-fold), isoorientin (5.6-fold), and nobiletin (2.5-fold). Kaempferol-3-O-neohesperidoside, kaempferol-3-O-robinobioside (biorobin), kaempferol-7-O-glucoside, luteolin-7-O-rutinoside, luteolin-4′-O-glucoside, luteolin-3′-O-glucoside and hispidulin were common across the three comparison groups. Luteolin-C-rhamnosyl-glucoside and quercetin-3-O-glucoside remained at similar concentrations from 6 to 9 days. In contrast, luteolin-7-O-glucoside (cynaroside), isosinensetin, pratensein, nobiletin, luteolin-6-C-glucoside (isoorientin), and diosmetin-7-O-galactoside increased from 6 to 9 days.

Some identified gene transcripts were likely associated with fruit quality. In the comparison of 3 days vs. 6 days, *peroxidase 72-like* (*POD*), *polyphenol oxidase* (*PPO*), *chlorophyllase-1* (*CHL1*), *linoleate 13S-lipoxygenase 2-1* (*LOX2-1*), *beta-amyrin 11-oxidase-like* and *chalcone synthase 2* (*CHS2*) were downregulated. In contrast, *histidine kinase 4-like* (*HK4*), *monogalactosyl diacylglycerol synthase 2* (*MGDG2*), *7-hydroxymethyl chlorophyll a reductase* (*HCAR*), *cytochrome P45090B1*, and *two-component response regulator ARR11* (*ARR11*) were upregulated. For metabolomics analysis of 3 days vs. 6 days, 2-hydroxyhexadecanoic acid, syringic aldehyde, and isorhamnetin-3-O-(6′-p-coumaroylglucoside) were increased. In the comparison of 6 days vs. 9 days, *9-cis-epoxycarotenoid dioxygenase* (*NCED2*), *peroxidase 27-like* (*POD27/POD66*), *LOX2-1*, *beta-carotene hydroxylase 2* (*CHY2*), *gibberellin 2-beta-dioxygenase 8-like protein* (*GB8*), *9-cis-epoxycarotenoid dioxygenase* (*NCED3*), *phenylalanine ammonia-lyase 5* (*PAL5*), *phenylalanine ammonia-lyase-like* (*PAL*), *zeaxanthin epoxidase* (*ZEP*), *probable carotenoid cleavage dioxygenase 4* (*CCD4*), *peroxidase 2-like* (*POD2*), and *carotenoid 9,10-cleavage dioxygenase 1* (*CCD1*) were increased, together with transcripts for the cell wall modifying enzymes *polygalacturonase* (*PG*) and *pectinesterase 2* (*PE*). *Peroxidase* (*POD*) is involved in cell wall lignin formation (Fig. 6F) and may be associated with chayote fruit texture. Other metabolites that also increased included lysoPC (20:3, 17:0, 17:1, 17:2, and 16:1), lysoPC (18:1, 17:0, 20:2, 20:3, 16:1, and 14:0) (2*n* isomer)*, lysoPE 15:1 and lysoPE (15:1, 16:1, 17:1, 18:1, 20:3, and 20:2) (2*n* isomer)*, which are associated with cell membrane structure^44^ and may be related to the rapid enlargement of chayote fruit. Comparison of the 3 days vs. 9 days transcriptome changes identified transcripts involved in cell wall structure and fruit texture, such as *expansin-like B1*, *glucan endo-1,3-beta-glucosidase 12*, *phenylalanine ammonia-lyase 5* (*PAL5*), and *glutamate dehydrogenase 2*, which were downregulated. Other gene transcripts, such as *POD2*, *ARR12*, *GB8*, *NCED2* and *NCED3*, also decreased. Several transcripts were increased, including *expansin-like B1*, which could be involved in inducing plant cell wall extension^45,46^, consistent with the rapid enlargement of chayote fruit. In addition, *HK4* and *CCD4*, which participate in ethylene signal transduction^47^ and flavonoid production^48^, respectively, also increased. Unsaturated acids were changed between the 3 days vs. 9 days metabolomes, besides flavonoids, isoflavones, lysoPC, and lysoPE.

Association analysis between the metabolome and transcriptome can aid in the understanding of transcriptional regulation mechanisms controlling metabolic pathways. According to this analysis, plant hormone signal transduction, alpha-linolenic acid metabolism, 2-oxocarboxylic acid metabolism, arginine biosynthesis, and glutathione metabolism were enriched in the 3 days vs. 6 days comparison, whereas flavonoid biosynthesis and accumulation were increased in the 6 days vs. 9 days comparison.

## Discussion

Chayote is popular for its appearance, nutrition and palatability and is a significant economic crop. This study reports the first high-quality genome assembly of 14 chayote chromosomes. The genome size is 606.42 Mb, which is similar to that of *L. cylindrica*^21^ (669 Mb) but smaller than those of *T. anguina*^37^ (919.8 Mb) and *B. hispida* (859 Mb)^23^. Other members of the Cucurbit have much smaller genomes, such as *C. sativus*^17^, *C. melo*^18^, *C. lanatus*^49^, *C. pepo*^20^, and *M. charantia*^50^. Approximately 401.08 Mb (65.94%) of repetitive sequences were predicted, which was less than the amount in the *B. hispida* genome and greater than that in the *C. melo* genome^18,23^. A total of 27,469 protein-coding genes were annotated, which is similar to the numbers in wax gourd and pumpkin^23,51^. This genome assembly will underpin further deep molecular-level research, trait selection, and evolutionary studies in Cucurbitaceae.

This study provides insights into WGD events and chayote evolution. WGD generates gene homologs and thus is of great significance during the diversification of species and the acquisition of new functions. As others predicted, Cucurbitaceae underwent four WGD events. First, cucurbit-common tetraploidization (CucWGD1 in Fig. 4C) occurred shortly after core-eudicot-common hexaploidy (ECH, 115–130 Mya)^52^. Second, pumpkin^51^ (*Cucurbita maxima* and *C. moschata*), zucchini^20^ (*C. pepo*) and silver-seed gourd^53^ (*Cucurbita argyrosperma*) were confirmed to have undergone a WGD event (CucWGD2 in Fig. 4C). Third, our data indicated that chayote underwent an additional WGD event at approximately 25 ± 4 Mya, denoted as CucWGD3 (Fig. 4C), which requires further exploration. Interestingly, the genome collinearity percentage between chayote and pumpkin was slightly higher than that between chayote and snake gourd (Fig. 5B). This may indicate that chayote generated more new genes, perhaps to adjust to a changed environment, after WGD occurred. A high degree of genome collinearity suggested that chayote contains conserved ancestral Cucurbitaceae genes, as is the case in pumpkin. Considerable changes have occurred during the evolution of cucurbitaceous species, as seen from the different shapes and lengths of chayote, snake gourd and towel gourd, which are closely related, according to the evolutionary tree (Fig. 4C).

Transcripts of genes affecting various metabolic pathways were found to increase during chayote fruit development in our study. A number of these factors affect pigment content, such as *HCAR* (7-hydroxymethyl chlorophyll a reductase), which, together with *CHL1*, is required for chlorophyll a synthesis^54^ and thus may affect chayote chlorophyll content. Several of the transcripts encoded enzymes that are involved in carotenoid metabolism. Beta-carotene hydroxylase (*CHY2*) can influence carotenoid content. *CCD1* and *CCD4* encode enzymes that cleave various carotenoids to yield β-ionone and α-ionone in other plants^48,55,56^ and are likely to affect fruit carotenoid content and flavor. It is probable that *NCED*2 and *ZEP* also participate in regulating chayote fruit pigments^57^ and abscisic acid biosynthesis^58^. Genes related to other hormones also showed increased expression, including *AUX, IAA* and *HK4*, which may be related to the major growth of chayote fruit^59^. Cellulose synthase-like protein B4 is associated with cell wall complexes^60^ and, together with polygalacturonase, pectinesterase may influence chayote cell wall properties and texture during fruit development. Furthermore, *13-LOX2-1* may be involved in chayote fruit defense^61^, although some LOX genes are also involved in flavor generation.

**Fig. 8:**
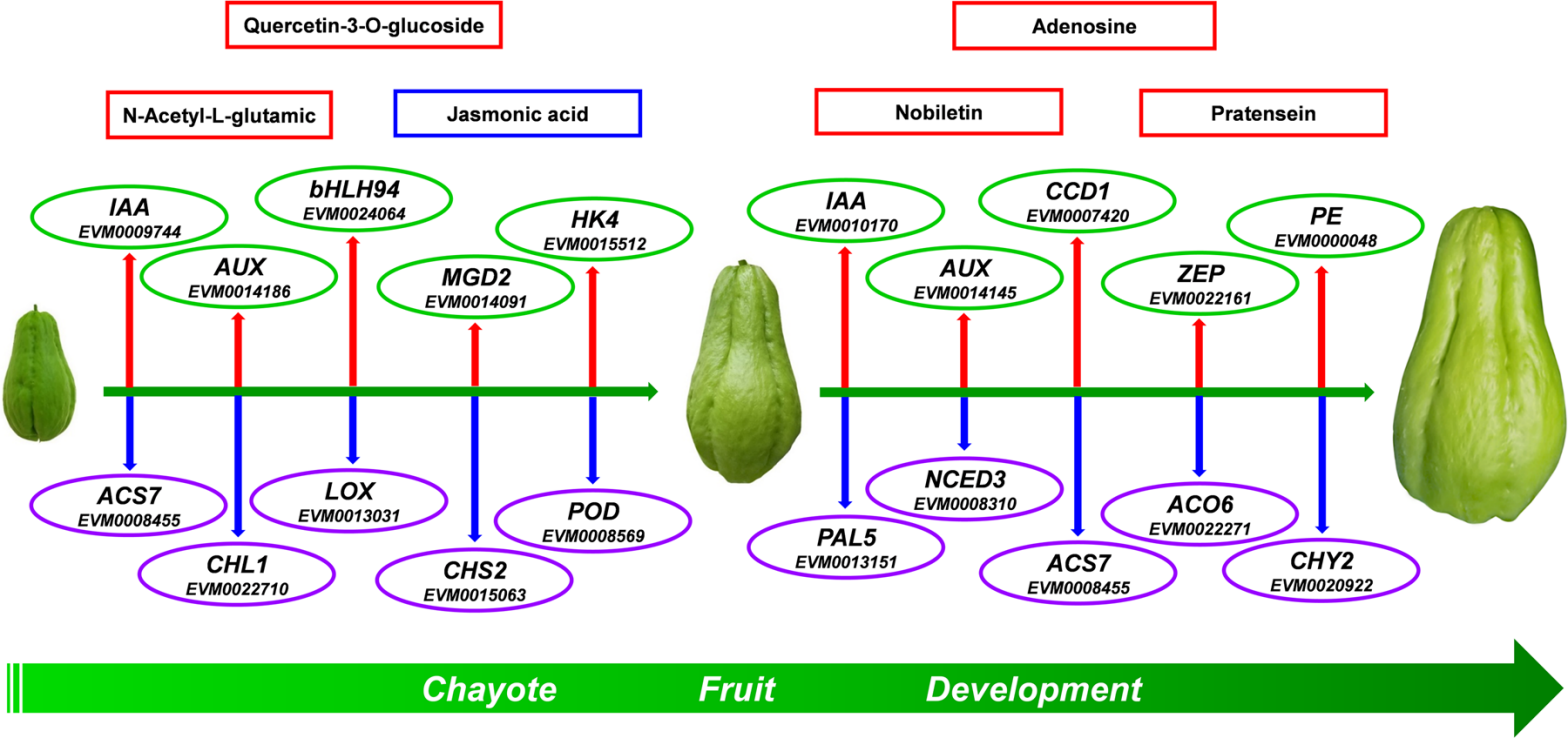
Schematic showing changes in transcripts during chayote fruit development. From left to right, fruit at 3, 6, and 9 days. The upward-facing arrows (red) indicate transcripts that increase with time. The downward-facing arrows (blue) represent transcripts that decrease with time. Metabolic components are circled in red (upregulated) or blue (downregulated)

Several isoflavonoid and flavonoid compounds increased in amount, such as nobiletin, hispidulin, isosinensetin, quercetin-3-O-rhamnoside, luteolin-4′-O-glucoside, luteolin-3′-O-glucoside and luteolin-7-O-rutinoside. Quercetin^62^, nobiletin and hispidulin are antioxidants with superoxide anion-scavenging activity, and their increased content in chayote may contribute important dietary health benefits^63,64^. A schematic showing major gene expression changes during chayote fruit development was constructed based on information from the transcriptome and metabolome (Fig. 8). Further in-depth investigation of the action of the genes identified in this study may explain specific aspects of chayote fruit texture, pigment, growth, flavor, and antioxidant properties.

## Materials and methods

### Chayote sample collection and genomic sequencing

Fresh chayote leaves were collected from Jianshui in Yunnan Province for genome sequencing. DNA was extracted from young chayote leaves, and two 350 bp libraries were constructed. The library was sequenced (150 bp reads at each end, PE150) using an Illumina sequencer. The raw information was assessed for GC distribution statistics and Q20 and Q30 quality value evaluations and then filtered to obtain clean reads that were used for genome size evaluation, genome assembly, GC content statistics, heterozygosity statistics, and post-assembly evaluation.

A Nanopore library was constructed and used for Nanopore third-generation sequencing in five steps: (1) large DNA fragment isolation, (2) fragment repair, (3) connecting reactions, (4) quantitative detection, and (5) library construction. Finally, single-molecule real-time sequencing was carried out on the PromethION sequencer to obtain the raw data prior to error correction to obtain high accuracy data. De novo genome assembly was performed by combining three strategies: initially, in the correction step, longer reads were selected by Canu^65^ (available at https://github.com/marbl/canu, v1.5) with the settings ‘genomeSize=1000000000’ and ‘corOutCoverage=50’, subsequently, overlapping raw reads were identified through the high-sensitivity overlapper MHAP (mhap-2.1.2, option ‘corMhapSensitivity=low/normal/high’), subsequently, error correction was performed by the falcon_sense method (option ‘correctedErrorRate=0.025’); followed by Smartdenovo, error correction using racon^66^ software (https://github.com/isovic/racon) and adjustment by Pilon^67^ software (v1.22, available at https://github.com/broadinstitute/pilon, with the parameters (‘--mindepth 10 --changes --threads 4 --fix bases’). The assembled results were assessed by evaluation of the ratio of the Illumina sequencing reads and the evaluation of BUSCO integrity. BUSCO^30^ v 2.0 (eudicotyledons_odb9 database) was used to validate the genome completeness and gene set completeness of the draft genome sequences.

Hi–C fragment libraries were constructed with 300–700 bp insert sizes and sequenced by the Illumina platform after five steps: (1) cell crosslinking, (2) endonuclease digestion, (3) end repair, (4) DNA cyclization, and (5) DNA purification and capture. Qubit 2.0 and Agilent 2100 instruments were used to detect library concentration and insert size. First, raw reads were trimmed, and low-quality PE reads were removed to obtain clean data. The clean Hi–C reads were aligned to the assembly results with bwa aligner^68^ (version: 0.7.10-r789) after a trim of clean reads at the putative Hi–C junctions. Only alignable read pairs with mapping quality greater than 20 were reserved for further analysis. Invalid read pairs, including dangling-end, self-circle, re-ligation, and “dumped” products, were filtered by HiC-Prov2.8.1. LACHESIS^69^ software was used for grouping, sequencing and orientation of genome sequences, and the assembly results were evaluated. Parameters for running LACHESIS were as follows: CLUSTER_MIN_RE_SITES, 64; CLUSTER_MAX_LINK_ DENSITY, 2; CLUSTER_NONINFORMATIVE_RATIO, 2; ORDER_MIN_N_RES_IN_TRUN, 15; and ORDER _MIN_N_RES_IN_SHREDS, 15.

### Gene prediction and function annotation

Based on the principles of structure prediction and de novo prediction, we constructed the repeat sequence database of the chayote genome through LTR_FINDER^70^ and RepeatScout^71^. A database of repeat elements in the chayote sequence was generated by PASTEClassifier^72^ and then merged with the Repbase database^73^ to generate the final database of repetitive sequences. The repetitive sequences of the chayote genome were predicted by RepeatMasker^74^ software based on the constructed repetitive sequence database.

Coding gene prediction analysis in the chayote scaffold sequences was carried out using three different methods: de novo prediction, homology-based species prediction and UniGene prediction. Then, EVM^73^ v1.1.1 software was used to integrate the prediction results. Genscan^75^, Augustus^76^ v2.4, GlimmerHMM^77^ v3.0.4, GeneID^78^ v1.4, and SNAP^79^ were used for *de nove* prediction; GeMoMa^80^ v1.3.1 was used for homologous species prediction; Hisat^81^ v2.0.4 and Stringtie^82^ v1.2.3 were used for assembly based on reference transcripts; TransDecoder v2.0 and GeneMarkS-T^83^ v5.1 were used for gene prediction; PASA^84^ v2.0.2 was used for the prediction of UniGene sequences without reference assembly based on transcriptome data; EVM v1.1.1 was used to integrate the prediction results obtained by the above three methods; and PASA v2.0.2 was used for modification. In addition, we also predicted different noncoding RNAs. Whole genome comparison and recognition of microRNAs and rRNAs were carried out with Blastn based on the Rfam^85^ database; tRNAs were identified by tRNAscan-SE^86^. Pseudogene prediction was also performed. Using the predicted protein sequence, through BLAT^87^ comparison, homologous gene sequences (possible genes) were identified in the genome, and we then used genewise^88^ to find premature termination codons and frameshift mutations in the gene sequences to identify the pseudogenes.

The predicted gene sequences were compared with NR^89^, KOG^90^, GO, KEGG^91^, TrEMBL^92^, and other functional databases. Databases were used to compare gene protein sequences by BLAST^93^ v2.2.31 (*E* value ≤ 1 × 10^−5^), and gene functions were annotated by KEGG pathway annotation analysis. KOG functional annotation analysis, GO functional annotation analysis, and other gene functional annotation analyses were carried out to obtain final annotations.

### Comparative analysis of genomes between species

Using OrthoFinder v2.3.7 software^94^, the protein-coding sequences in the chayote genome and genomes from 13 other species were compared. The PANTHER V15 database^95^ was used to annotate the obtained gene families. Finally, GO and KEGG enrichment analyses were carried out by using clusterProfiler v3.14.0^96^ to identify the gene families unique to chayote.

The evolutionary tree was constructed using the maximum likelihood method by IQ-TREE v1.6.11^97^ software and single-copy protein sequences, with the number of bootstrap replicates set to 1000. For the evolutionary tree, we set the outer group as *A. trichopoda* to obtain a rooted tree. MCMCTREE, a software package in PAML v4.9i^98^, was used to calculate the divergence time. The number of Markov chain iterations was set as follows: burnin 500000, sampfreq 10, nSample 5000000.

According to the evolutionary tree results with differentiation time and gene family clustering by CAFE v4.2 (Computational Analysis of gene Family Evolution) software^99^, the number of gene family members of each branch’s ancestors was estimated by a birth mortality model to predict the contraction and expansion of gene families of species relative to their ancestors (*p* < 0.05). We identified the expanded and contracted gene families in chayote and annotated them with PANTHER. GO and KEGG enrichment analyses were carried out with clusterProfiler.

### WGD event and collinearity analysis

Diamond v0.9.29.130^100^ was used to compare the gene sequences between two species and identify similar gene pairs (*E* value < 1 × 10^−5^, *C* score > 0.5, JCVI software^101^ was used to filter the *C* score). Then, according to the gff3 document, MCScanX^102^ was used to determine whether similar gene pairs are adjacent on chromosomes and finally, collinear gene blocks were obtained.

WGDs are events in which the genome is doubled. At present, the Ks (synonymous mutation rate) method and 4DTv (fourfold synonymous third-codon transversion rate) methods are commonly used to identify WGDs; here, wgd v1.1.0 software^103^ and a custom script (https://github.com/JinfengChen/Scripts) were used to identify WGD events in chayote.

### Transcription analysis

Fresh chayote fruit samples at 3, 6, and 9 days were collected for transcription analysis, and three biological replicates were performed for each stage. The cDNA libraries were obtained by polymerase chain reaction (PCR) enrichment. After the library was checked for quality by Q-PCR, the Illumina platform was used for sequencing. Low-quality and adapter reads were removed to obtain clean data, which were used for sequence alignment with the specified reference genome. The transcriptome was assembled using StringTie^82^. Differential expression analysis was performed between the different sample groups. Pearson’s correlation coefficient (*r*) was used for repeatability assessment^104^. DESeq2^105^ was used for differential expression analysis between sample groups to obtain the DEG sets between two biological samples. Hierarchical clustering analysis was carried out for the screened DEGs to find groups of genes with the same or similar expression patterns. For the detection of DEGs, fold change > 2 and false discovery rate (FDR) < 0.01 were used as cutoff values. As a screening standard, fold change (0.01) represents the FDR between two samples with a corrected *p* value for significant differences. For functional annotation and enrichment analysis of DEGs, gene function annotation was performed as described for gene prediction and functional annotation above.

### Metabolites analysis

Freshly collected chayote fruit samples were used for metabolome analysis. The freeze-dried samples were crushed into powder prior to ultra performance liquid chromatography–mass spectrometry (MS)/MS^106,107^ analysis. Analyst 1.6.3 software was used to process mass spectrum data. Based on the local metabolic database, the metabolites contained in the samples were quantitatively and qualitatively analyzed by MS^108,109^. To determine the repeatability of metabolite extraction and detection, one QC sample was inserted per ten samples. The total ion current diagram was overlapped and analyzed. Principal component analysis^110^, cluster analysis^96^ and Spearman rank correlation (*R*) were used for metabolome data assessment. OPLS-DA^111^ analysis was performed for each difference group. Subsequently, the differential multiple, *p* value of the *t* test and VIP value of the OPLS-DA model were combined to screen differentially accumulated metabolites^111^. The screening criteria were FC > 2, *p* value < 1 and VIP > 1.

### Transcriptome and metabolism conjoint analysis

The results of metabolome analysis were combined with the results of transcriptome analysis, and the DEGs and differentially accumulated metabolites in the same group were simultaneously mapped to the KEGG pathway map^112^. Genes and related metabolic pathways were identified for analysis and data screening using a *p* value < 0.05 to identify significant relationships.

## Supplementary information

Appendix

Table S6 Transcriptome data

Table S7 Metabolomic data

## Data Availability

The chayote raw genome and transcriptome sequencing data are available from the NCBI under project ID PRJNA640239.
