# Supplementary material for: Combined genomic, transcriptomic, and metabolomic analyses provide insights into chayote (Sechium edule) evolution and fruit development
Source: Hortic Res. 2021 Jan 31;8:35. doi: 10.1038/s41438-021-00487-1 (PMC7847470; doi:10.1038/s41438-021-00487-1)
Supplement: Supplementary file 1 — Appendix [file 41438_2021_487_MOESM1_ESM.docx]

**Appendix**

**Supplemental Table 1 Hi-C Assembly data statistics**

| **Group** | **Cluster Number** | **Cluster Length(bp)** | **Order Number** | **Order Length(bp)** |
| --- | --- | --- | --- | --- |
| LG01 | 71 | 72,434,893 | 65 | 71,556,316 |
| LG02 | 53 | 53,388,938 | 47 | 52,829,034 |
| LG03 | 37 | 48,596,451 | 30 | 47,890,124 |
| LG04 | 18 | 48,121,734 | 16 | 47,910,514 |
| LG05 | 13 | 46,657,517 | 12 | 46,560,561 |
| LG06 | 52 | 47,620,171 | 42 | 46,595,483 |
| LG07 | 27 | 44,288,647 | 22 | 43,717,866 |
| LG08 | 33 | 40,726,058 | 30 | 40,447,277 |
| LG09 | 36 | 41,247,058 | 30 | 40,599,363 |
| LG10 | 31 | 38,359,683 | 27 | 37,889,988 |
| LG11 | 30 | 36,829,293 | 23 | 36,143,760 |
| LG12 | 18 | 35,027,922 | 16 | 34,791,988 |
| LG13 | 29 | 27,930,660 | 15 | 26,593,742 |
| LG14 | 11 | 25,193,477 | 9 | 24,949,239 |
| Total(Ratio %) | 459(97.04) | 606422502(99.71) | 384(83.66) | 598475255(98.69) |

**Supplemental Table 2 Post-Hi-C genome statistics**

| **Parameter** | **Number/Length** |
| --- | --- |
| Scaffold number | 103 |
| Scaffold length (bp) | 608,211,883 |
| Scaffold N50 (bp) | 46,561,661 |
| Scaffold N90 (bp) | 26,595,142 |
| Scaffold max (bp) | 71,562,716 |
| Gap total length (bp) | 37,000 |
| Contig number | 473 |
| Contig length (bp) | 608,174,883 |
| Contig N50 (bp) | 8,395,526 |
| Contig N90 (bp) | 500,000 |
| Contig max (bp) | 24,203,044 |
| GC content (%) | 38.71 |

**Supplemental Table 3 Repeat sequence statistics**

| **Type** | **Number** | **Length** | **Rate(%)** |
| --- | --- | --- | --- |
| ClassI | 732,812 | 355,943,362 | 58.52 |
| ClassI/DIRS | 25,442 | 22,115,169 | 3.64 |
| ClassI/LARD | 331,335 | 142,092,214 | 23.36 |
| ClassI/LINE | 33,426 | 7,123,603 | 1.17 |
| ClassI/LTR/Copia | 147,290 | 55,432,158 | 9.11 |
| ClassI/LTR/Gypsy | 157,873 | 169,396,150 | 27.85 |
| ClassI/LTR/Unknown | 9,803 | 2,042,809 | 0.34 |
| ClassI/PLE | 2,002 | 725,521 | 0.12 |
| ClassI/SINE | 16,667 | 2,833,477 | 0.47 |
| ClassI/TRIM | 8,424 | 2,798,084 | 0.46 |
| ClassI/Unknown | 550 | 144,745 | 0.02 |
| ClassII | 39,075 | 13,955,023 | 2.29 |
| ClassII/Crypton | 8 | 464 | 0.00 |
| ClassII/Helitron | 1,906 | 223,632 | 0.04 |
| ClassII/MITE | 2,748 | 419,814 | 0.07 |
| ClassII/Maverick | 137 | 248,443 | 0.04 |
| ClassII/TIR | 29,859 | 12,471,560 | 2.05 |
| ClassII/Unknown | 4,417 | 685,691 | 0.11 |
| PotentialHostGene | 25,657 | 4,344,684 | 0.71 |
| SSR | 8,295 | 1,621,846 | 0.27 |
| Unknown | 151,836 | 59,331,350 | 9.76 |
| Total | 957,675 | 401,077,680 | 65.94 |

**Supplemental Table 4 Statistical of gene prediction**

| **Method** | **Software** | **Species** | **Gene number** |
| --- | --- | --- | --- |
| Ab initio | Genscan | - | 24,923 |
|  | Augustus | - | 31,321 |
|  | GlimmerHMM | - | 63,096 |
|  | GeneID | - | 36,476 |
|  | SNAP | - | 37,414 |
| Homology-based | GeMoMa | Arabidopsis_thaliana. TAIR10 | 25,441 |
|  |  | Citrullus_lanatus | 28,605 |
|  |  | Cucumis_sativus.ChineseLong | 27,974 |
|  |  | Luffa_cylindrica | 29,929 |
| Aseq | TransDecoder | - | 51,475 |
|  | GeneMarkS-T | - | 32,597 |
|  | PASA | - | 48,646 |
| Integration | EVM | - | 28,237 |

**Supplemental Table 5 Statistical of genetic information**

|  | **Number** | **Length(bp)** |
| --- | --- | --- |
| Gene | 28,237 | 136,904,950 |
| Average gene | -- | 4,848.42 |
| Exon | 160,955 | 47,455,214 |
| AveExon | 5.70 | 1,680.60 |
| CDS | 154,866 | 35,547,024 |
| Average CDS | 5.48 | 1,258.88 |
| Intron | 132,718 | 89,449,736 |
| Average Intron | 4.70 | 3,167.82 |

**
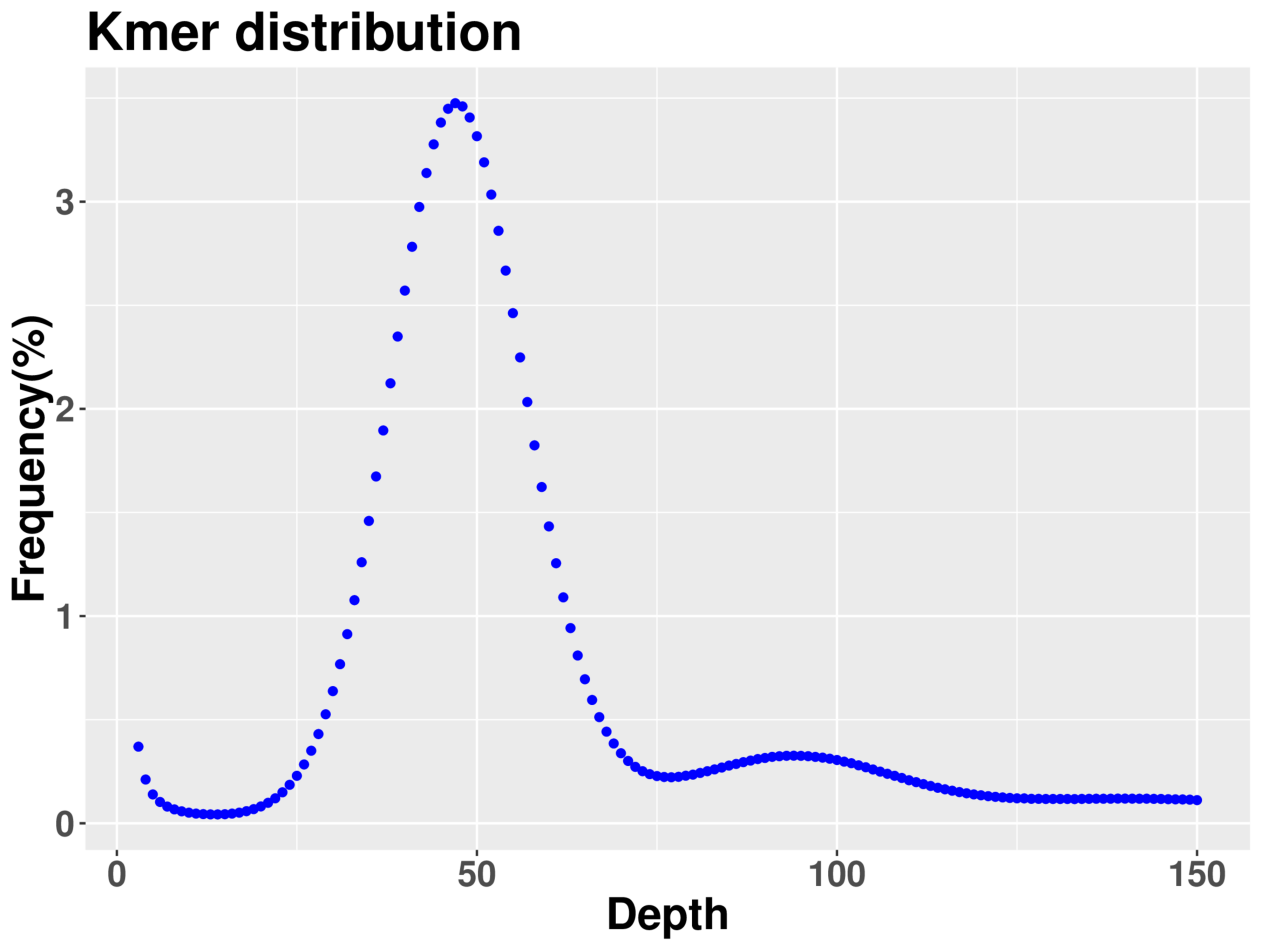
**

**Supplemental Figure 1 K-mer (k=19) distribution.**

**
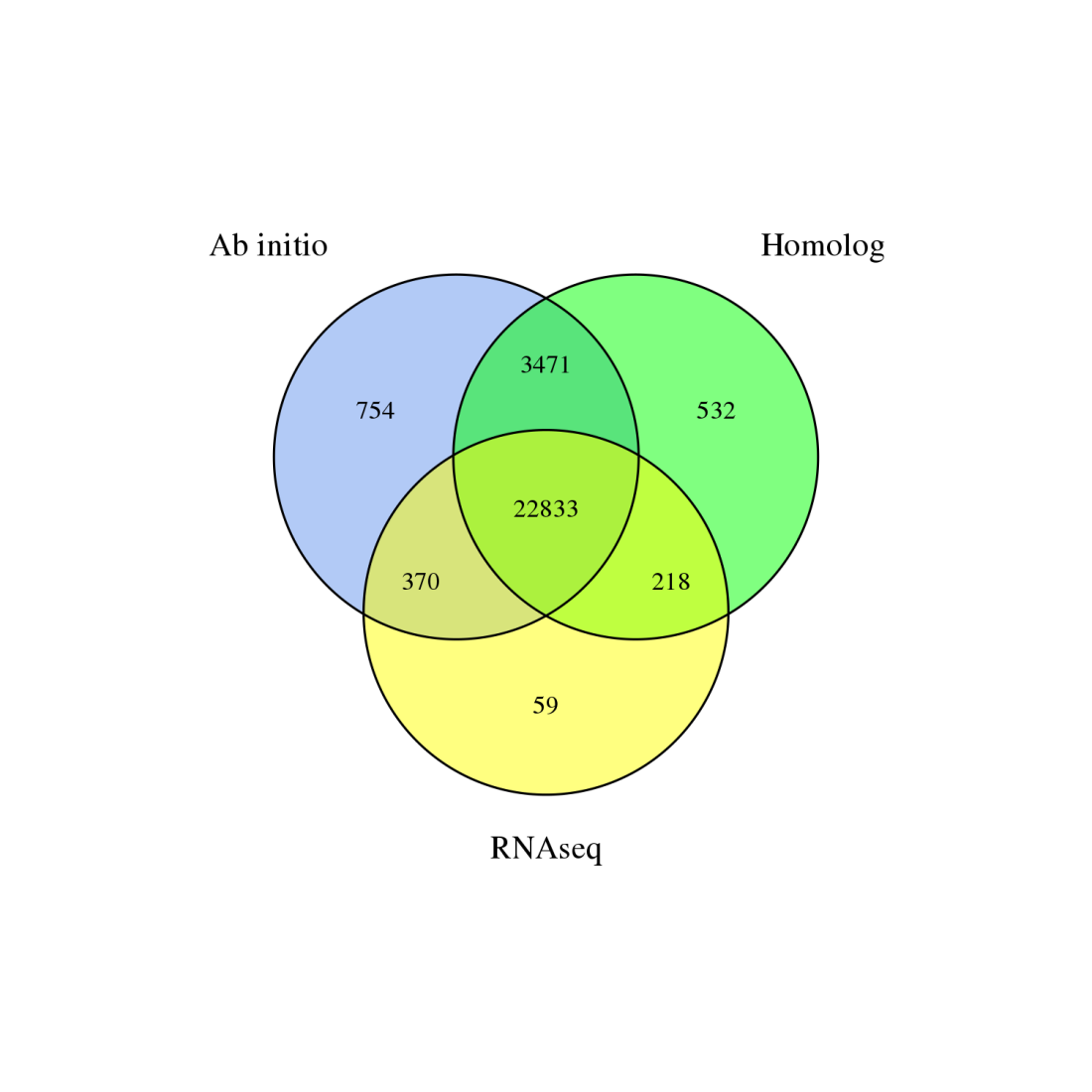
**

**Supplemental Figure 2 Genes derived from the distribution map of the three prediction methods.**

**
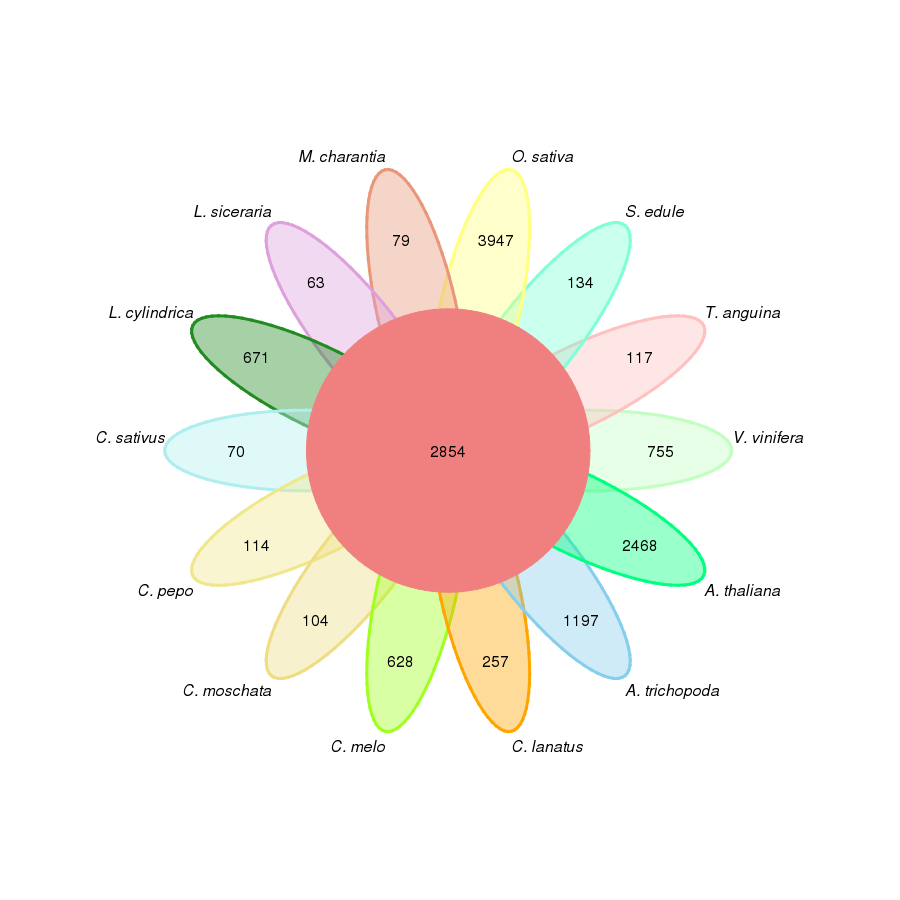
**

**Supplemental Figure 3 Gene family cluster petal diagram.**

**
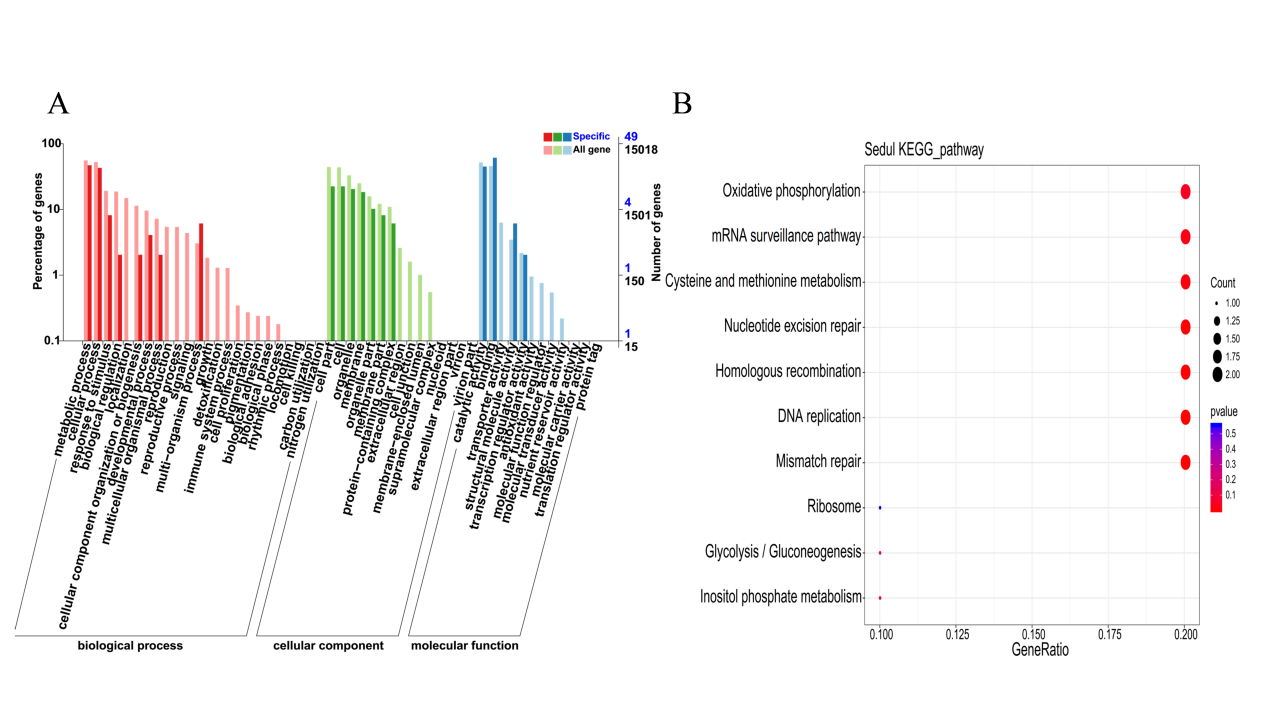
**

**Supplemental Figure 4 A****(GO) and B(KEGG) annotation of chayote specific gene families.**

**
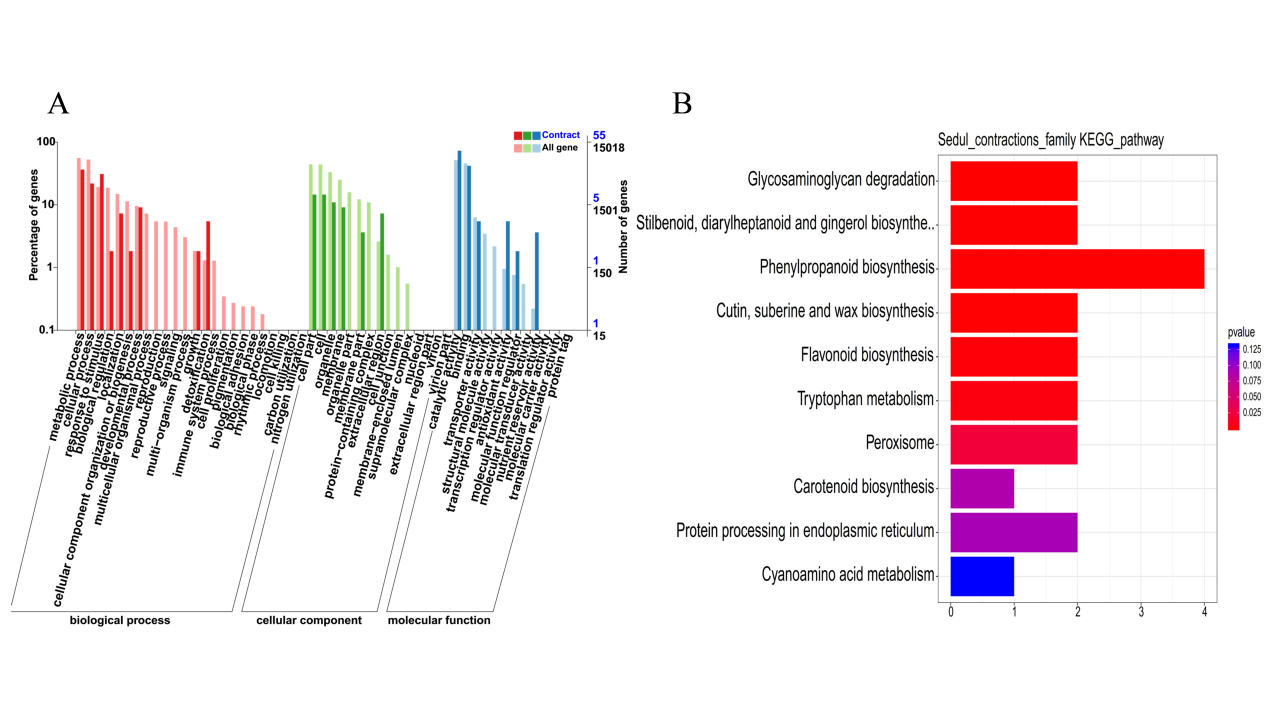
**

**Supplemental Figure 5 A(GO) and B(KEGG) annotation of chayote contraction families.**

**
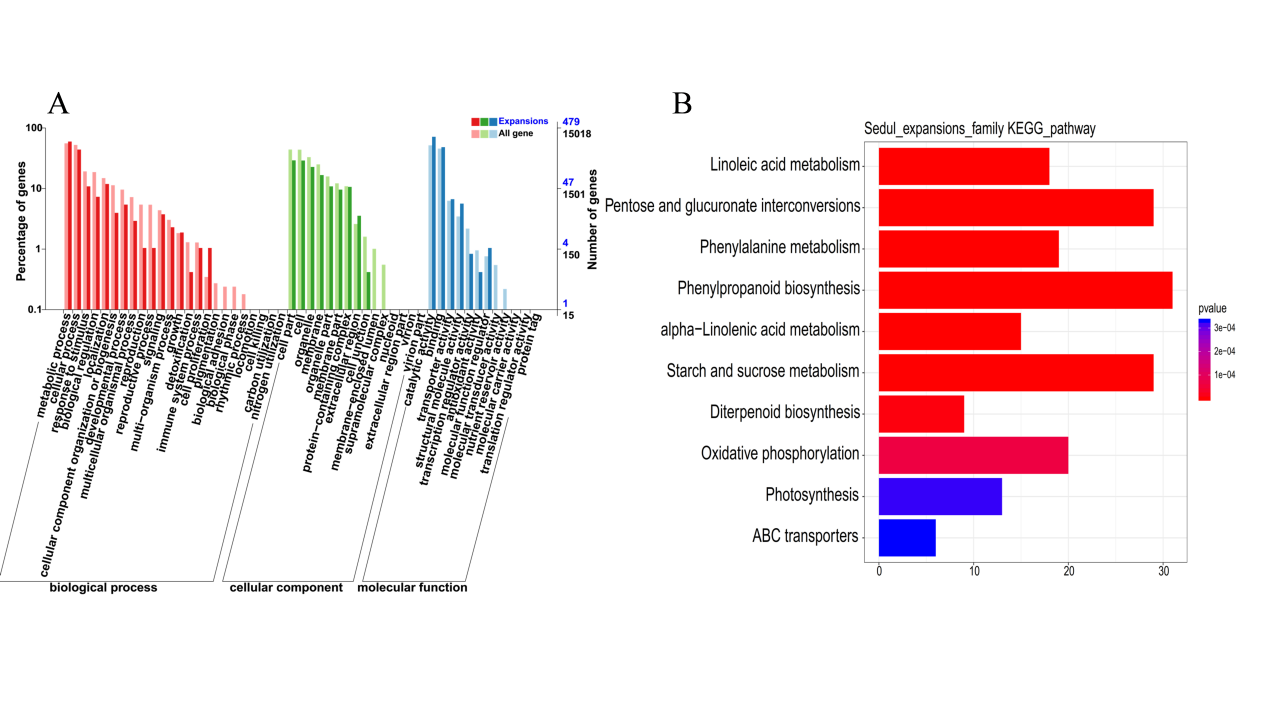
**

**Supplemental Figure 6 A(GO) and B(KEGG) annotation of chayote expansion families.**


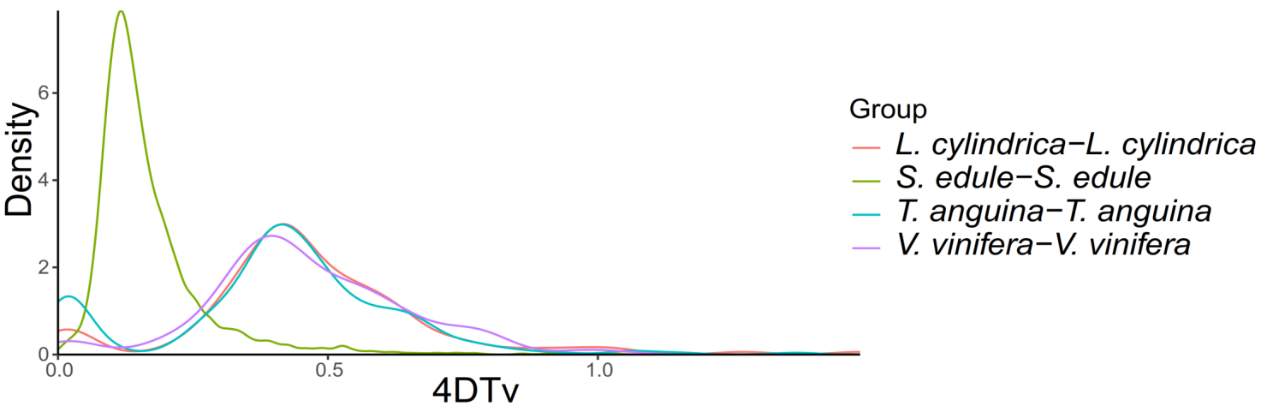


**Supplemental Figure 7 4DTv drawing of chayote and other comparative plant species.**

**
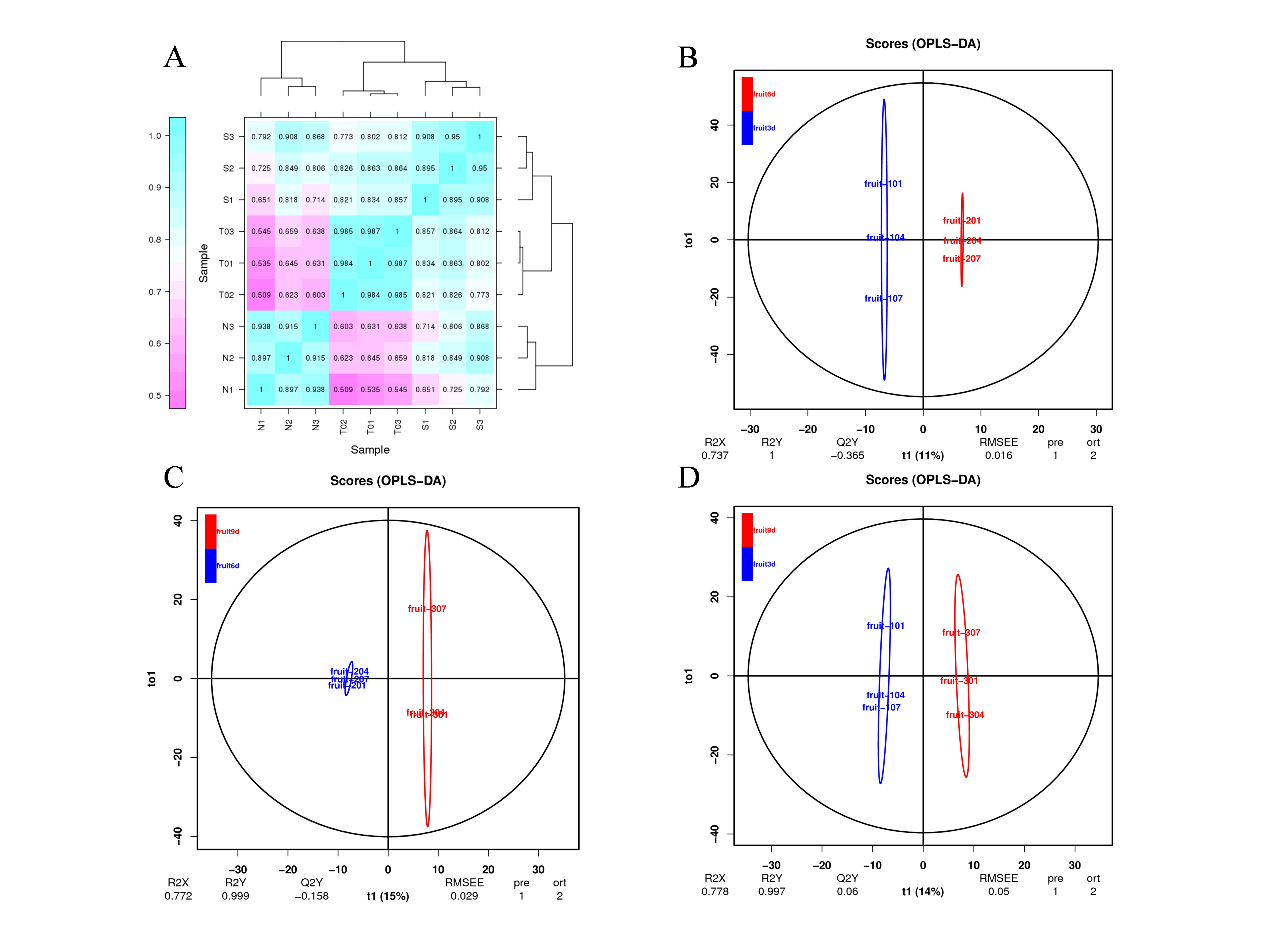
**

**Supplemental Figure 8 A: Correlation between samples in transcriptomics; B: OPLS-DA metabonomic assessment in 3d vs 6d; C: OPLS-DA metabonomic assessment in 6d vs 9d; D: OPLS-DA metabonomic assessment in 3d vs 9d.**


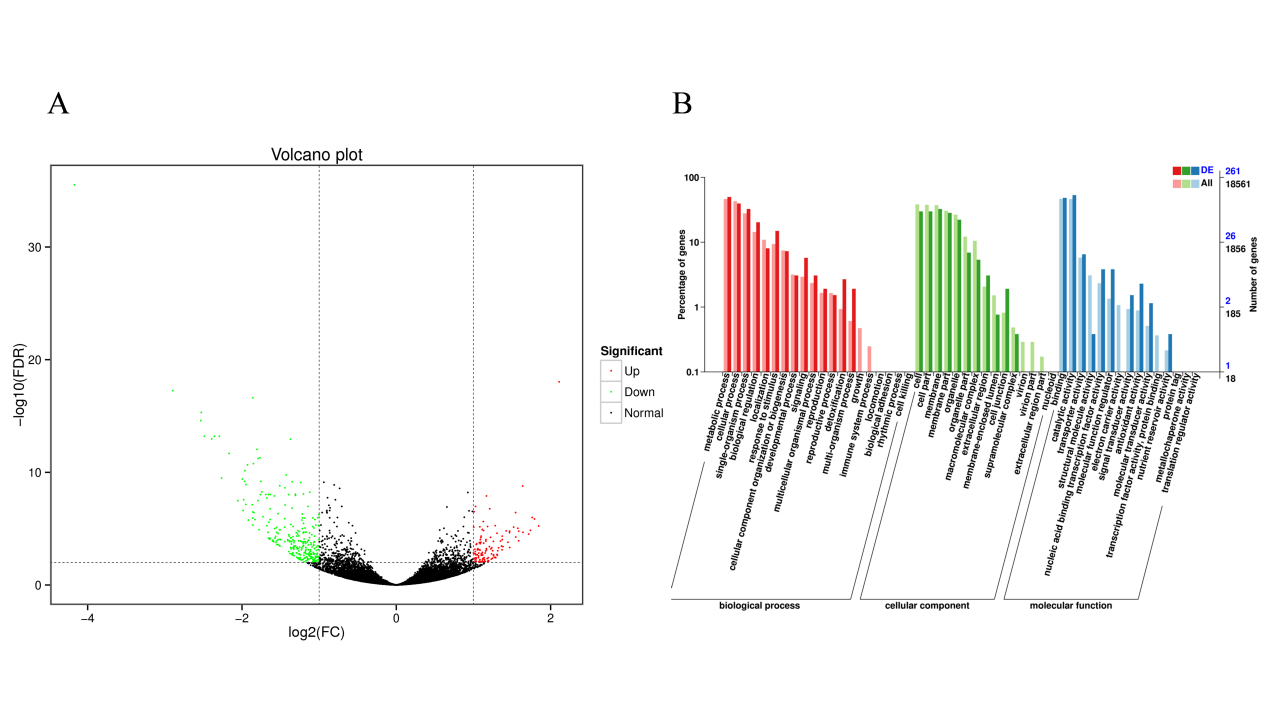


**Supplemental Figure 9 DEGs** [**transcriptome**](C:/Users/Administrator/AppData/Local/youdao/dict/Application/8.9.3.0/resultui/html/index.html#/javascript:;) **analysis of chayote in 3d vs 6d group. A: Volcano diagram of DEGs; B: GO annotation of DEGs.**

**
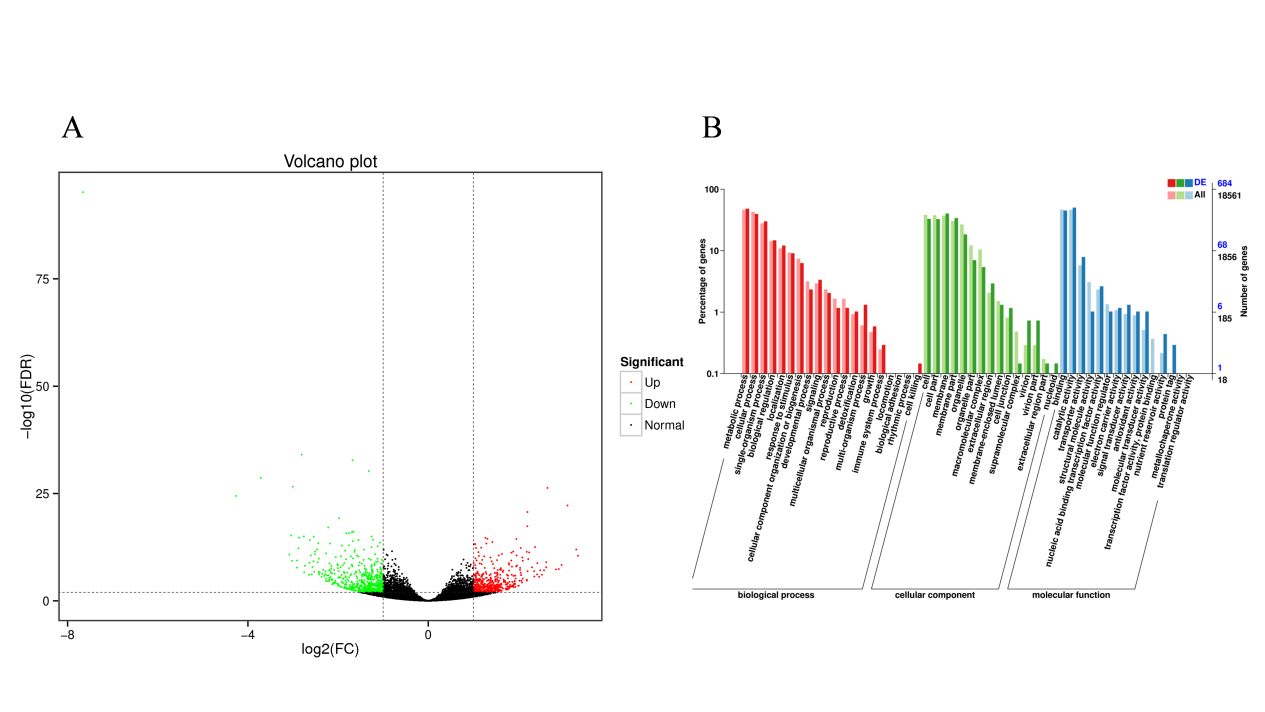
**

**Supplemental Figure 10 DEGs** [**transcriptome**](C:/Users/Administrator/AppData/Local/youdao/dict/Application/8.9.3.0/resultui/html/index.html#/javascript:;) **analysis of chayote in 6d vs 9d group. A: Volcano diagram of DEGs; B: GO annotation of DEGs.**

**
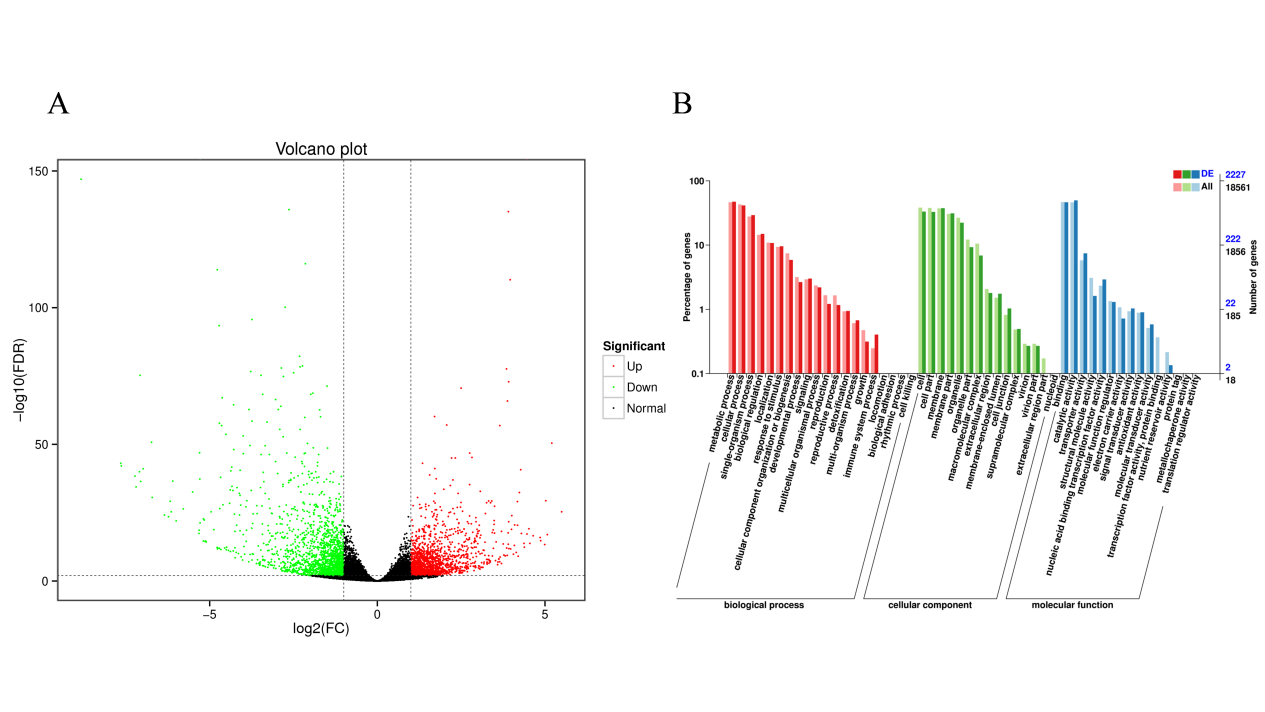
**

**Supplemental Figure 11 DEGs** [**transcriptome**](C:/Users/Administrator/AppData/Local/youdao/dict/Application/8.9.3.0/resultui/html/index.html#/javascript:;) **analysis of chayote in 3d vs 9d group. A: Volcano diagram of DEGs; B: GO annotation of DEGs.**

**
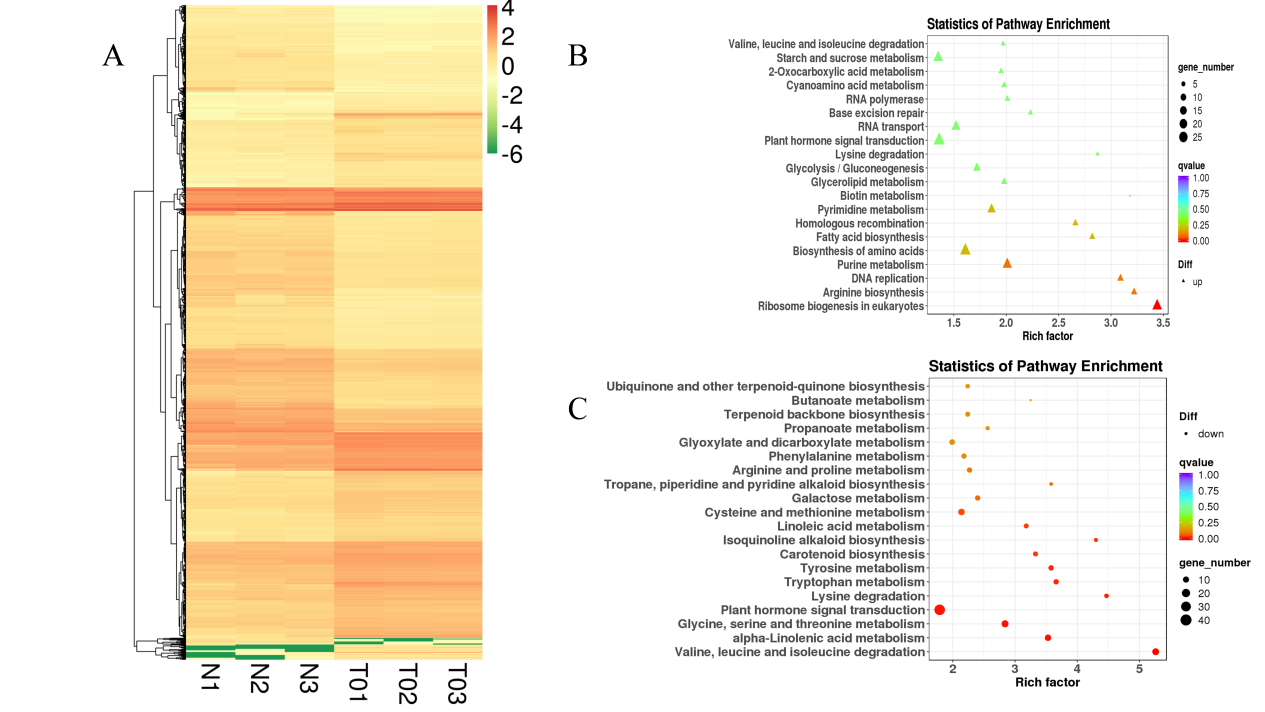
**

**Supplemental Figure 12 DEGs** [**transcriptome**](C:/Users/Administrator/AppData/Local/youdao/dict/Application/8.9.3.0/resultui/html/index.html#/javascript:;) **analysis of chayote in 3d vs 9d group. A: Cluster heat map of DEGs; B: KEGG annotation of up-regulated DEGs. C: KEGG annotation of down-regulated DEGs.**
